# Strategies of Anode Design for Seawater Electrolysis: Recent Development and Future Perspective

**DOI:** 10.1002/smsc.202200030

**Published:** 2022-08-16

**Authors:** Tanveer ul Haq, Yousef Haik

**Affiliations:** ^1^ Sustainable Energy Engineering Frank H. Dotterweich College of Engineering Texas A&M University Kingsville TX 78363-8202 USA; ^2^ Department of Mechanical and Nuclear Engineering University of Sharjah Sharjah UAE

**Keywords:** anode designs, electronic structures, hydrogen production, nanomaterials, seawater electrolysis

## Abstract

Compared with freshwater splitting, seawater electrolysis has more spaces to be explored, which is primarily attributed to the additional critical catalytic challenges of the competition between anodic oxygen evolution reaction (OER) and chlorine chemistry, deep corrosion, and site blocking due to the presence of chloride ions and insoluble particulate in seawater. However, if direct seawater electrolysis can be realized, it would revolutionize the energy and environmental sectors. In this review, the current effective strategies are summarized, including electronic modulation, oxygen vacancies creation, amorphous and porous structure design, corrosion‐resistant passive layer decoration, and creating strong catalyst–support interactions. The review also provides insights for seawater electrolysis on rational design of the OER catalyst with high selectivity, activity, corrosion resistance, chemical, and mechanical durability. Beyond the progress made to date, a perspective in the fabrication of high‐performance anodes for direct seawater electrolysis is also proposed. Collectively, this review will shed light on the rational design of a viable anode for massive and sustainable hydrogen fuel production from immense seawater.

## Graphical abstract

An overview of recent strategies for the rational design of anode material to overcome the selectivity, reactivity, durability, and corrosion resistance challenges in direct seawater electrolysis is offered in this review. The design strategies include electronic modulation, oxygen vacancies creation, amorphous and porous structure design, corrosion‐resistant passive layer decoration, and the creation of strong catalyst–support interactions.

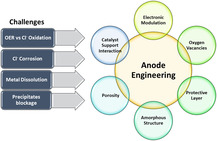

## 1 Introduction

The expanded usage and rapid depletion of traditional fossil fuels create energy and environmental crises and need sustainable alternatives. Hydrogen (H_2_) is deemed a potential choice due to its high energy density by mass (142 MJ kg^−1^) and potential for decarbonization across diverse sectors.^[^ ^1^ ^]^ According to a recent report, the demand for global H_2_ is anticipated to escalate to $ 100 billion in 2022.^[^ ^2^ ^]^ Currently, 95% of total H_2_ is generated from steam reforming of fossil fuels, accountable for massive CO_2_ emissions (830 Mt year^−1^).^[^ ^3^ ^]^ Exploration of renewable energy resources like water is the potential candidate for a sustainable and safe future because it can be converted to green fuel (H_2_) via electrocatalysis and can be used similarly to fossil fuels.^[^ ^4^ ^]^ Water splitting (to break the O─H bond) is an energy‐intensive process.^[^ ^5^ ^]^ Researchers have been practicing numerous technologies for electrochemical H_2_ production to reduce the cost and increase efficiency, that is, alkaline water electrolysis, anion exchange membrane water electrolysis, proton exchange membrane water electrolysis, and solid oxide electrolysis.^[^ ^6^ ^]^ The cathodic and anodic heterogeneous reactions in which hydrogen and oxygen evolution occur are common in all modules.^[^ ^7^ ^]^ Both electrochemical reactions entail the diffusion of water and removal of gases molecules at triple phase through adsorption–desorption of intermediates.^[^ ^8^ ^]^ The hydrogen evolution reaction (HER) has fast kinetics due to fewer intermediates, and many cost‐effective electrocatalysts have been fabricated for cathodic reactions.^[^ ^9^, ^10^ ^]^ A bottleneck is the multielectron, multistep oxygen evolution reaction (OER) process, which demands high activation potential for O─O bond formation and retards the rate of overall water electrolysis.^[^ ^11^, ^12^ ^]^

**Figure 1:**
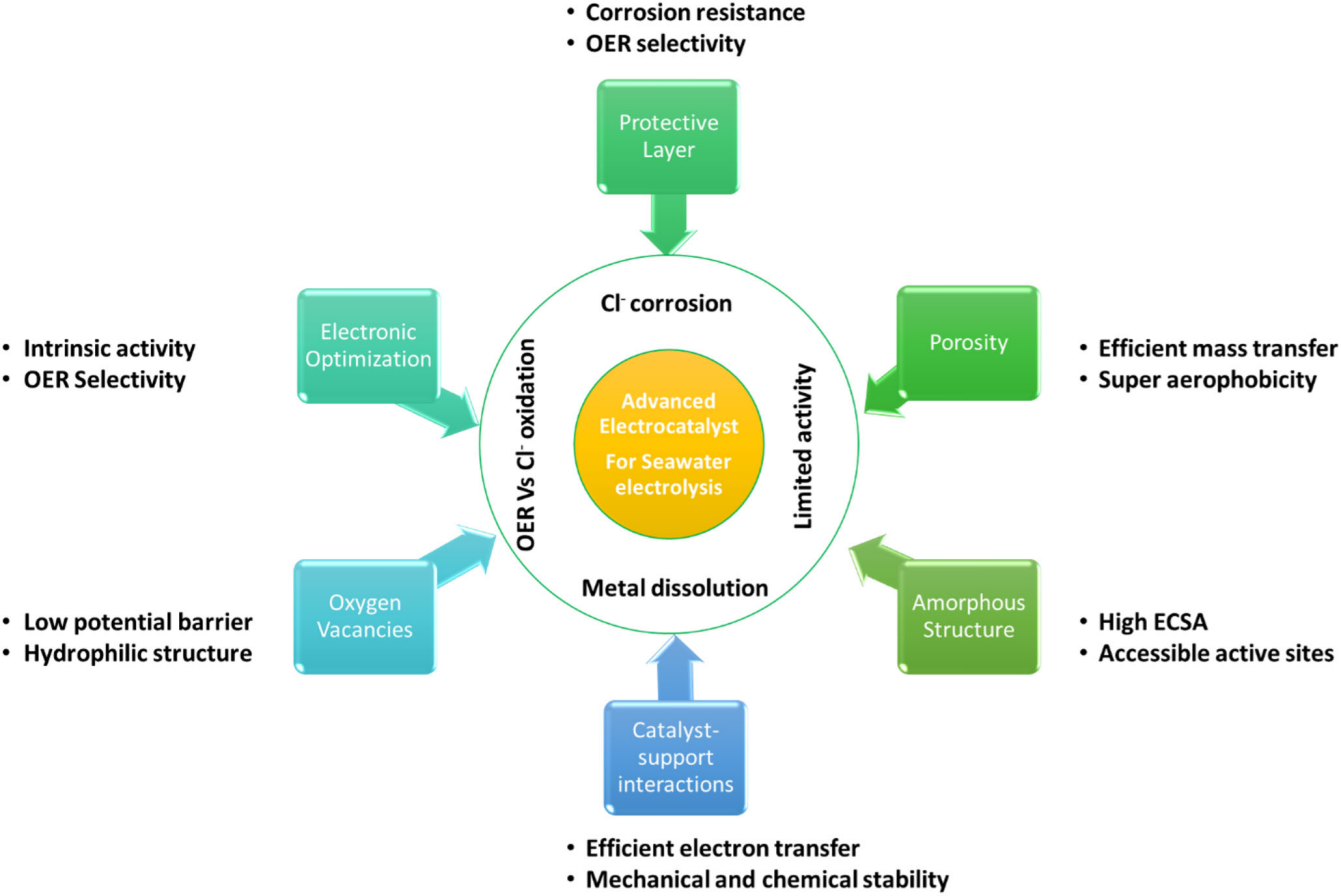
Scheme for anode characteristics to improve efficiency of direct seawater electrolysis.

Currently, fresh and purified water is used for electrolysis, but its resources are limited for many communities worldwide, and most of the population is at high risk of freshwater security.^[^ ^13^ ^]^ The purity of water feeds is attained by the pretreatment purification process using an external desalination plant or by integrating the electrolyzer system with the water purification system. These purification/desalination processes impose considerable economic constraints due to the investment cost of land, plantation, maintenance, and transportation. Direct seawater electrolysis has the potential to remove the pretreatment system, simplify the engineering of electrolyzers, and enhance the economic viability of green hydrogen production from water. The replacement of freshwater with abundant seawater in electrolyzers is highly advisable because oceans and seawater correspond to 97% of the Earth's total water.^[^ ^14^ ^]^ It is also facile to integrate renewable power generation technologies (e.g., wind, solar, and wave) installed in the coastal zone with seawater electrolysis.^[^ ^15^ ^]^ Moreover, it has also revealed that seawater electrolysis is significant for arid zone or coastal hyperarid as this technology can produce safe and highly pure drinking water.^[^ ^16^ ^]^ However, it is extremely challenging to achieve this task due to multiple challenges. The first challenge is the competition between water oxidation and chloride anion oxidation at the anode surface. Although the electrochemical oxidation of Cl^−^ anions produced precious products, that is, hypochlorite, hypochlorous acid, and Cl_2_ gas, they alter the solution conditions and cause the severe corrosion of electrodes.^[^ ^17^ ^]^ Hence electrodes should be selective for OER to sustain their performance. The second challenge is the presence of different non‐innocent ions in seawater that further hampers the OER kinetics.^[^ ^18^ ^]^ The third bottleneck in seawater electrolysis is the lack of durability of the electrocatalyst due to chloride corrosion, the presence of microbes (such as bacteria) in actual seawater, and the formation of insoluble precipitates (e.g., Mg(OH)_2_, Ca(OH)_2_).^[^ ^19^ ^]^ A way to use natural seawater as feedstock is to develop selective, efficient, and sustainable electrode material appropriate for unpurified water.^[^ ^20^ ^]^ The strategies to ameliorate the performance of an electrocatalyst are enhancing the number and intrinsic activity of exposed active sites.^[^ ^21^ ^]^ Different methods have been developed to improve the intrinsic activity of each active site, that is, defect engineering, phase engineering, multimetals, and crystal facet engineering.^[^ ^22^ ^]^ While nanostructuring, hybridization of active sites with conductive support is used to expose more active sites.^[^ ^23^ ^]^ These strategies work together to attain the highest performance and work well in freshwater electrolysis, but electrodes should have more advanced features to perform electrochemical reactions efficiently in aggressive seawater. These features are passive layer formation on the electrocatalyst surface to increase corrosion resistance, electronic modulation, oxygen deficiencies to enhance the selectivity of anode material for OER, porosity to accelerate the seawater diffusion, and gas evolution for industrial‐level turnover frequency. The amorphous structure improved the efficient charge transfer and fast kinetics, while the active sites’ growth on the conductive 3D substrate increases the conductivity and structural sustainability for a long lifetime.^[^ ^24^ ^]^ These requirements should be satisfied together and incorporated into one electrocatalyst to make this seawater electrolysis viable for massive H_2_ production (**Figure** **1**).

Here, we review the bottlenecks and recent innovative strategies for developing advanced anode material, aiming to directly use unpurified seawater in the electrolysis process. Beyond the emergent research, remaining challenges and emerging solutions with personal perspectives are also discussed. It is anticipated that this review will open up more endeavors into the rational design of electrodes for economic and massive H_2_ production from seawater.

## 2 OER Mechanism in Alkaline Conditions

In basic electrolyte, water is reduced to hydroxyl ions (OH^−^) and hydrogen molecule (H_2_) (Equation (1)).

(1) $$2H_{2}O+2e^{-}\to H_{2}+2{\text{OH}}^{-}$$

The following reaction occurs on the anode (Equation (2))

(2) $$4{\text{OH}}^{-}\to O_{2}+2H_{2}O+4e^{-}$$

The OER is multielectron, multistep process with high activation barriers, and each step accounts for a different type of loss (e.g., voltage and ohmic loss). The below equations (Equation ((3), (4), (5), (6), (7), (8))) demonstrate the various proposed mechanisms for O–O coupling which is given below.

(3) $$M+{\text{OH}}^{-}\to M-\text{OH}+e^{-}$$

(4) $$M-\text{OH}+{\text{OH}}^{-}\to M=O+e^{-}+H_{2}O$$

(5) $$M=O+{\text{OH}}^{-}\to M-\text{OOH}+e^{-}$$

(6) $$M-\text{OOH}+{\text{OH}}^{-}\to M+H_{2}O+e^{-}+O_{2}$$

(7) M=O+M=O→M−O−O−M

(8) $$M-O-O-M\to 2M+O_{2}$$

**Figure 2:**
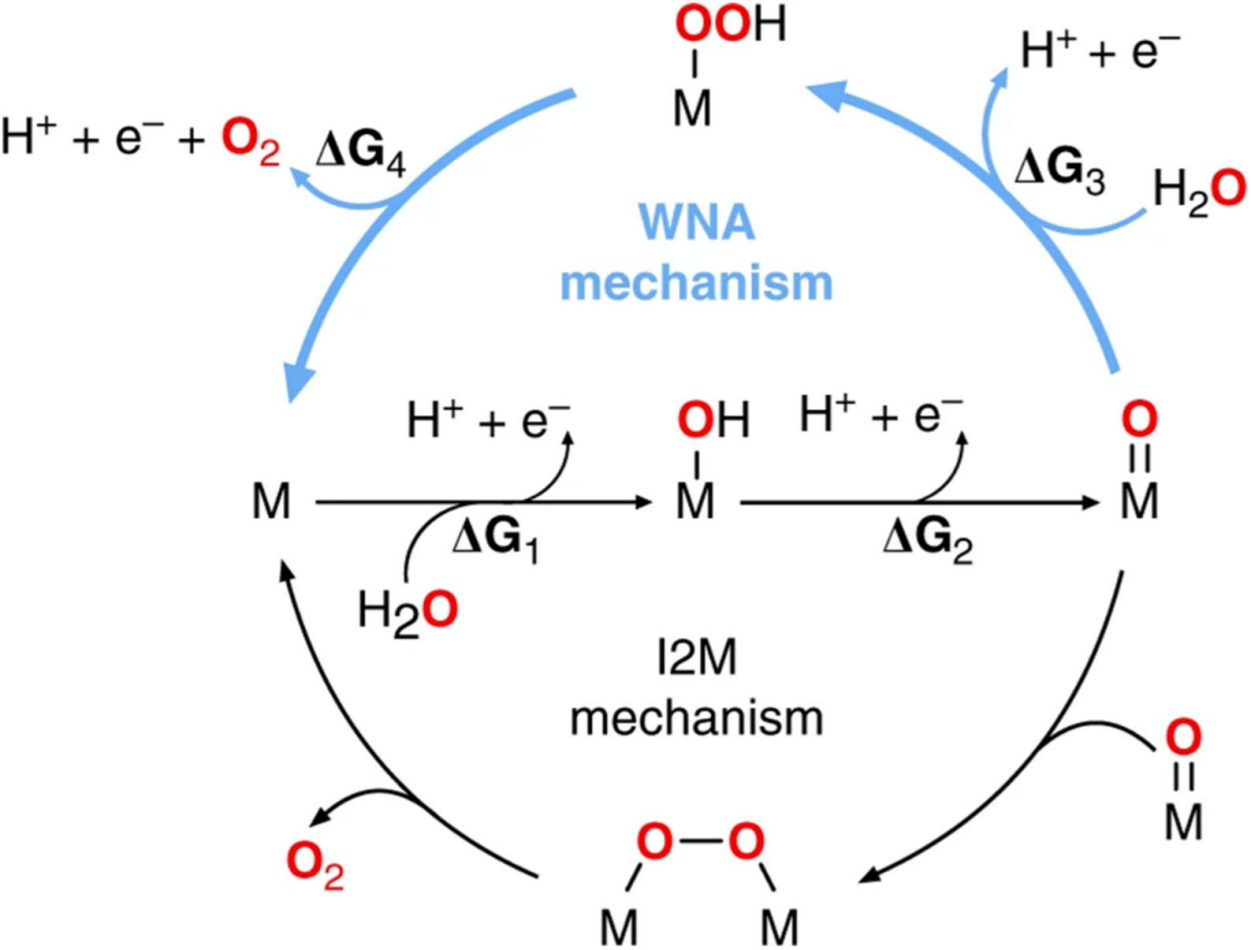
OER mechanism. a) Single‐site mechanism and b) dual‐site mechanism. Reproduced under the terms of the CC‐BY 4.0 license.^[^ ^25^ ^]^ Copyright 2019, The Authors, published by Springer Nature.

Equation ((3), (4), (5), (6)) follows the dual‐site mechanism where active sites participate in O–O coupling and support the water oxidation reaction.^[^ ^25^ ^]^ While in a single‐site mechanism (Equation ((3), (4), (7), (8))) one active site is involved in the four‐concerning proton‐coupled electron transfer process and triggers the O─O bond formation via oxyhydroxide intermediate formation, which is considered a high activation barrier step in OER (**Figure** **2**).^[^ ^26^ ^]^

Recent findings suggested that the bifunctional mechanism remarkably reduces the activation barrier for O–O coupling step. In a bifunctional mechanism, the single site mediates the O─O bond formation through the highest occupied molecular orbital (HOMO) and lowest unoccupied molecular orbital (LUMO) interaction of hydroxyl ions and M═O intermediate, where the adjacent acceptor site accepts the protons in the concerted reaction (Equation (9)).^[^ ^27^ ^]^

(9) $$M=O+{\text{OH}}^{-}+A\to M+A-H+O_{2}+e^{-}$$

Besides the discrepancies in the O–O coupling route, it is widely recognized that the M─O bond in the intermediate plays a crucial role in regulating the M active center's activity. The M─O bond strength can be used as a descriptor for OER selectivity and activity; a too weak M─O bond precludes the intermediate binding. At the same time, a too strong bond decreases the desorption rate of gaseous products and reduces the contact portion between active sites and electrolyte molecules. The chemisorption energy or Gibbs free energy for each step in OER is expressed as follows (Equation ((10), (11), (12), (13))).

(10) $$\Delta G_{1}=\Delta G\left({\text{OH}}^{*}\right)-\Delta G\left(*\right)+k_{b}T\text{ln}a_{H}^{+}-eU$$

(11) $$\Delta G_{2}=\Delta G\left(O^{*}\right)-\Delta G\left({\text{OH}}^{*}\right)+k_{b}T\text{ln}a_{H}^{+}-eU$$

(12) $$\Delta G_{1}=G\left({\text{OOH}}^{*}\right)-\Delta G\left(O^{*}\right)+k_{b}T\text{ln}a_{H}^{+}-eU$$

(13) $$\Delta G_{1}=\Delta G\left(*\right)-\Delta G\left({\text{OOH}}^{*}\right)+k_{b}T\text{ln}a_{H}^{+}-eU$$

Each step involved the removal of electron (oxidation process), and Δ*G* is the related Gibbs free energy for each step. While Δ*G* (*), Δ*G* (OH^*^), Δ*G* (O^*^), and Δ*G* (OOH^*^) are the chemisorption energy (Gibbs free energy) of the pristine active center and OH, O, and OOH intermediate adsorbed on the surface, respectively, eU is the shift in electron transfer under applied anodic potential, *k*_b_ is the Boltzmann constant (1.38 × 10^−23 ^m^2^Kg s^−2^ k^−1^), *a* ^+^_H_ represents the proton activity, and *T* is room temperature (*T* = 298.15 K).^[^ ^25^ ^]^ For an ideal catalyst the potential barrier related to the chemisorption energy of each step is equally spaced and needs the same amount of energy in each step: Δ*G*_1_ = Δ*G*_2_ = Δ*G*_3_ = Δ*G*_4_. However, experimental findings demonstrated that the Gibbs free energy related to the peroxide formation (OOH) is higher than the other step and is in the following order Δ*G*_3_ > Δ*G*_2_ = Δ*G*_1_ > Δ*G*_4_.^[^ ^28^ ^]^ It is found that peroxide formation is the rate‐determining step due to the electron‐dense nature of MO surface that inhibits the attack of OH^−^. The improvement in the chemisorption energy of this intermediate to reduce the potential barrier helped the performance and kinetics of OER electrocatalyst.

## 3 Challenges in Seawater Electrolysis

**Figure 3:**
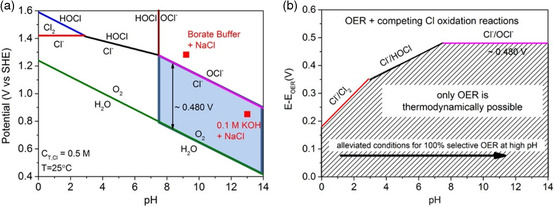
a) The Pourbaix diagram for simulated seawater containing 0.5 m NaCl aqueous solution where the absence of other chlorine sources ensured that the total chlorine species is 0.5 m. The water oxidation electrode potential has also been included, presuming oxygen partial pressure equal to 0.021 MPa. Two red points demonstrate the applied potential needed for Ni‐Fe LDH to deliver the geometric activity of 10 mA cm^−2^ after 1 h chronopotentiometry in 0.1 m KOH with 0.5 m NaCl (pH 13; lower right side) and 0.3 m borate buffer with 0.5 m NaCl (pH 9.2; upper right side). The area colored with light blue shows the proposed design criterion. b) The kinetic overpotential limit for OER catalyst as a fundction of pH has been calculated from the thermodynamic Pourbaix diagram to get 100% OER selectivity. The three possible Cl^−^ oxidation reactions have been considered to obtain these standard potential values. The dashed area indicated the thermodynamic selectivity of OER while above the line Cl^−^ oxidation reactions have been thermodynamically favorable. Reproduced with permission.^[^ ^30^ ^]^ Copyright 2016, Wiley‐VCH.

The electro‐oxidation of Cl^−^ anions comprises complicated reactions depending on the electrolyte's applied potential, operating temperature, and pH values. The disproportionation of hypochlorite ions (ClO^−^) and partial dissociation of Cl_2_ further complicates the electrochemistry of Cl^−^ ions.^[^ ^29^ ^]^ Peter Strasser and his co‐workers computed a Pourbaix diagram for aqueous Cl^−^ at room temperature by fixing the mass density of Cl^−^ to 0.5 m (average Cl^−^ mass in seawater) (**Figure** **3**).^[^ ^30^ ^]^ In acidic electrolyte (pH < 3), the chlorine evolution reaction (CER) is favorable over other oxidation reactions of Cl^−^ (Equation (14)).

(14) $$2{\text{Cl}}^{-}\to {\text{Cl}}_{2}+2e^{-}(\text{pH}=0,E^{0}=+1.36V_{\text{SHE}})$$

The Pourbaix diagram demonstrated that the CER is thermodynamically less favorable over OER, but the standard potential for CER is slightly higher than that for OER. In addition, CER is a single‐step two‐electron process and has much faster kinetics than multi‐intermediate, multielectron OER reaction.

In recent findings, researchers established three different mechanistic routes for Cl–Cl coupling, that is, Volmer–Heyrovsky, Volmer–Tafel, and Krishtalik (Equation ((15), (16), (17), (18), (19))).^[^ ^31^ ^]^

Volmer reaction

(15) $$2{\text{Cl}}^{-}+2^{*}\to {\text{Cl}}^{*}+e^{-}+{\text{Cl}}^{-}$$

Heyrovsky reaction

(16) $${\text{Cl}}^{*}+{\text{Cl}}^{-}\to *+{\text{Cl}}_{2}+e^{-}$$

Tafel reaction

(17) $${\text{Cl}}^{*}+{\text{Cl}}^{*}\to 2^{*}+{\text{Cl}}_{2}$$

Krishtalik reaction

(18) $${\text{Cl}}^{*}+{\text{Cl}}^{-}\to {\text{Cl}}^{*+}+e^{-}+{\text{Cl}}^{-}$$

(19) $${\text{Cl}}^{*+}+{\text{Cl}}^{-}\to *+{\text{Cl}}_{2}$$

The first step involves the adsorption of Cl^−^ ions on active sites (Cl^*^) and is common in all three pathways. The Heyrovsky route comprises the direct recombination of Cl^−^ on the adsorbed Cl^*^ while in the Tafel pathway, two adsorbed chlorine sites recombine for Cl–Cl coupling when the distance between two active centers is less than the Vander wall radii of Cl_2_. In the Krishtalik route, chloronium ions recombine with Cl^−^ ions, and Cl_2_ gas desorbs from the active sites.

In an alkaline environment (pH > 8), hypochlorite formation (ClO^−^) is the central reaction (Equation (20)).

(20) $${\text{Cl}}^{-}+2{\text{OH}}^{-}\to {\text{ClO}}^{-}+H_{2}O+2e^{-}(\text{pH}=14,E^{0}=+0.89V_{\text{SHE}})$$

The thermodynamic potential difference between two competing reactions (OER and Cl^−^ oxidation) increases with increasing pH. Compared with neutral and acidic conditions, an alkaline system can exhibit large kinetic overpotential for selective water oxidation. In an alkaline environment, the equilibrium potential for ClO^−^ formation is around 0.48 V higher than water oxidation. In other words, the OER catalyst must operate below this kinetic overpotential for maximum selectivity and avoid Cl^−^ chemistry interference. In addition to the alkaline design criterion to maximize the equilibrium potential difference between two competing reactions, few other strategies have been established to increase the selectivity of anode material for OER. These strategies are 1) development of hybrid material with Cl^−^ blocking layer next to the OER active sites to repel the diffusion of chloride ions from the electrolyte and 2) engineering of active sites to increase its selectivity for OH^−^ intermediates by optimizing the surface and potential energy to attain the desired Faradic efficiency (FE).^[^ ^29^ ^]^ The commonly observed overpotential for OER is higher than CER due to multielectrons involved in the mechanism and makes water oxidation reaction kinetically unfavorable.^[^ ^32^ ^]^ Therefore, it is highly demandable to develop active, sustainable, and selective anode material to avoid the generation of toxic and corrosive Cl^−^‐related compounds during seawater electrolysis. In seawater splitting, the HER and OER are associated with local pH variation at the electrode and regarded as a critical issue.^[^ ^33^ ^]^ During electrolysis, there has been a continuous reduction in pH at anode due to OH^−^ oxidation and an increment in pH at cathode due to H^+^ reduction. Studies revealed that this pH fluctuation is in 5–9 pH units and triggers severe catalyst degradation even at a small geometric activity.^[^ ^34^ ^]^ The continuous decrease in pH in the anodic chamber increases the acidity near the electrode surface and causes severe anode corrosion. The pH increment at the cathode vicinity decomposes the bicarbonate ions (HCO_3_^−^) present in seawater to carbonate (CO_3_^2−^) ions. These carbonate ions hydrolyze and can lead to the precipitation of magnesium hydroxide Mg(OH)_2_ (Equation (21)).^[^ ^35^ ^]^

(21) $${\text{MgCO}}_{3}\left(s\right)+H_{2}O\left(l\right)\to \text{Mg}{\left(\text{OH}\right)}_{2}\left(s\right)+{\text{CO}}_{2}\left(g\right)$$

The precipitates block the electrode surface and cause the mass and ion diffusion limitation. The Pourbaix diagram unveiled that the pH of seawater should be >7.5 for selective OER and to prevent the Cl^−^ ion interruption. Although the trace content of carbonate and borate in seawater can act as a buffer, their limited capacity does not change the situation. Stabilization of pH variation can only be viable using supporting electrolytes.^[^ ^36^ ^]^

The experimental findings revealed that seawater's aggressive Cl^−^ ions could severely erode the active sites and current collectors and convert the active metal sites to the corresponding hydroxide via the metal chloride–hydroxide formation process. The Cl^−^ etching/corrosion mechanism comprises three steps, that is, polarization, dissolution, and hydrolyzation.^[^ ^37^ ^]^ At high anodic potential, the HOMO–LUMO orbital energy of anode is at the lower energy level, triggering the massive Cl^−^ ion adsorption to the positively polarized active surface. In the absence of a passive layer, these aggressive anions coordinate with the adsorbed ions and cause dissolution. The OH^−^ ions in an alkaline environment accelerate the hydroxide formation from metal chloride ions. The etching mechanism comprises the following three reactions successively (Equation ((21), (22), (23))).

(22) $$\text{Absorption}:\text{ M}+{\text{Cl}}^{-}\to {\text{MCl}}_{\text{ads}}+e^{-}$$

(23) $$\text{Dissolution}:{\text{ MCl}}_{\text{ads}}+{\text{xCl}}^{-}\to {\text{MCl}}_{x}^{-}$$

(24) $$\text{Hydroxide formation}:{\text{ MCl}}_{x}^{-}+x{\text{OH}}^{-}\to M{\left(\text{OH}\right)}_{x}+x{\text{Cl}}^{-}$$

**Figure 4:**
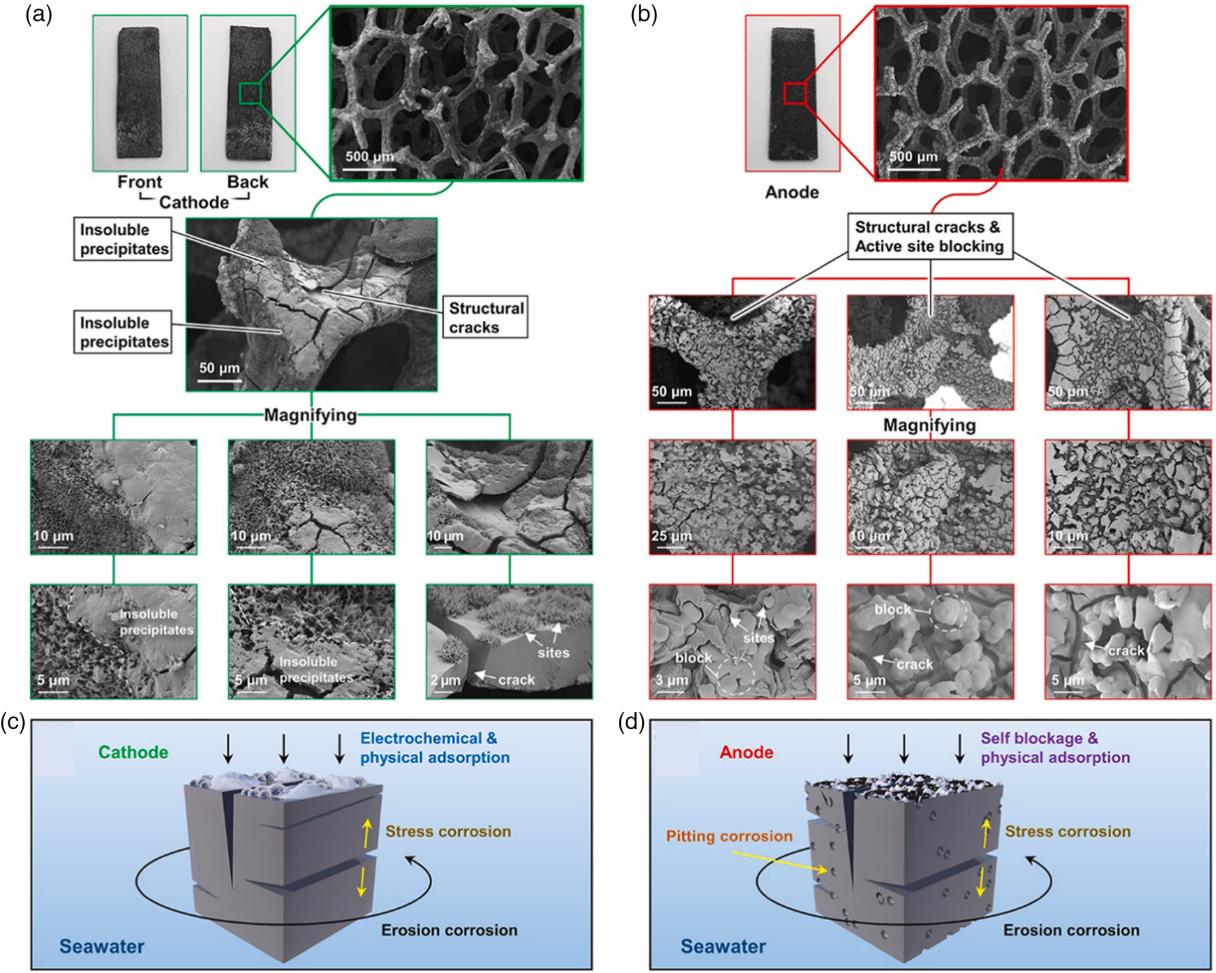
a,b) Scanning electron microscopy (SEM) images of Co/Ni‐doped defect‐rich Cu‐based oxides and Ni/Co‐doped defect‐rich Cu‐based sulfides after long‐term electrolysis in natural seawater. The postcharacterization reveals the white residue embedded in the interior part of the cathode and covering layer and structural cracks on the anode surface. c,d) The systematic models for cathode and anode, respectively, have shown the OER degradation mechanism. Reproduced with permission.^[^ ^38^ ^]^ Copyright 2021, Elsevier.

In addition to Cl^−^ ions, there are plenty of noninnocent anions and cations, small particulates, and microbes/bacteria, which poison the active sites and limit electrode materials’ long‐term sustainability (**Figure** **4** **)**.^[^ ^38^ ^]^ These ions and particulates also impart severe issues to the membrane used to separate cathode and anode. Despite these bottlenecks, direct seawater electrolysis has demonstrated outstanding potential for massive H_2_ fuel production with innovative technology and material development strategies.

## 4 Rational Design of Anode Material for Seawater Oxidation

Recently, extensive efforts have been devoted to fulfilling the demands of industrial seawater electrolysis. The following sections summarize the recent progress in advanced features of anode materials to tackle the challenges mentioned above in seawater electrolysis. Five advanced features of anode materials termed electronic modulation, oxygen vacancies, passive layer, amorphous and porous structure, and strong catalyst–support interactions are explained, emphasizing integrating all the above features into one electrocatalyst for supporting aggressive seawater electrolysis with high efficiency and corrosion resistance.

### 4.1 Electronic Optimizations

**Figure 5:**
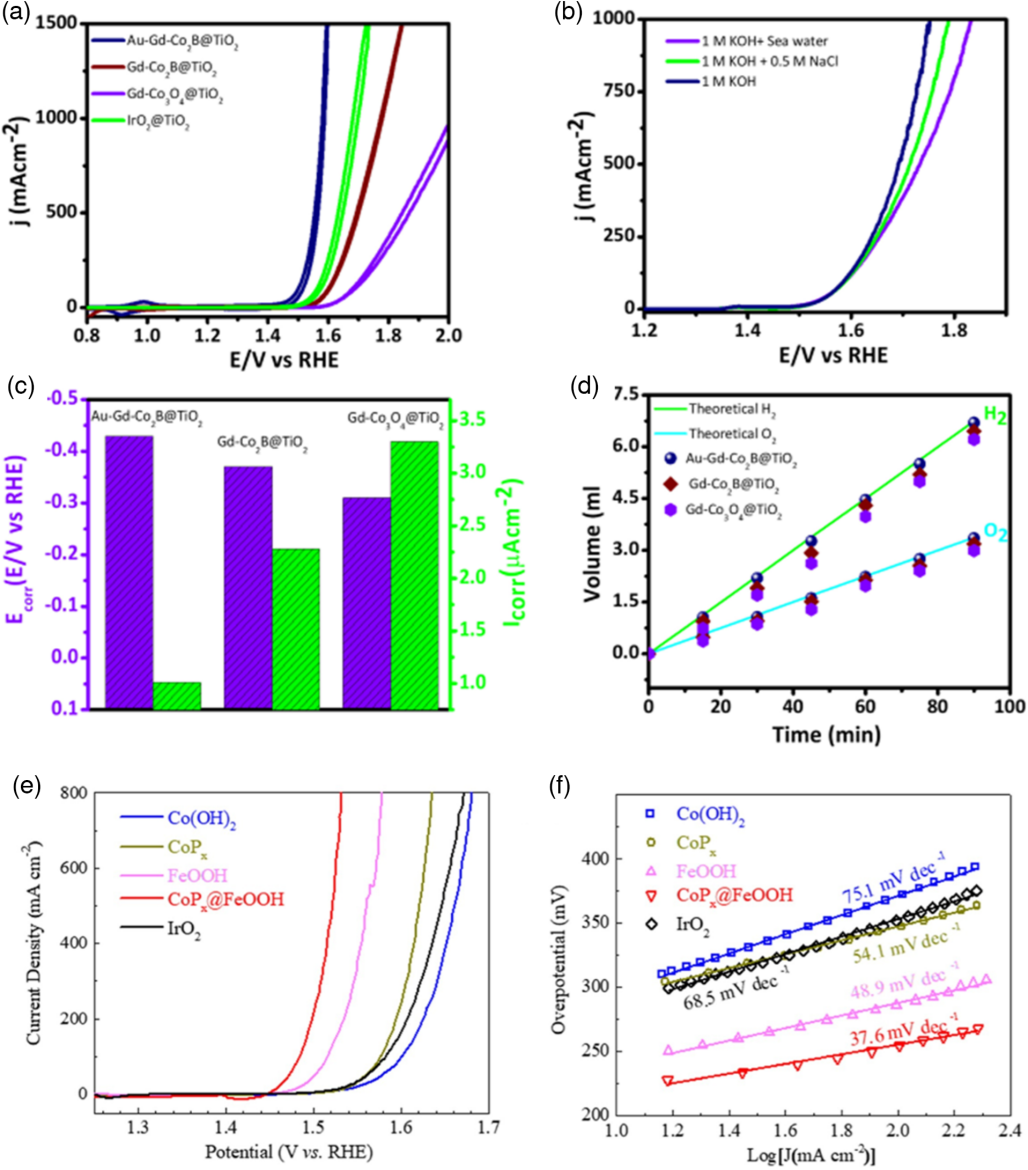
Au–Gd–Co2B@TiO_2_ catalyst for seawater splitting. a,b) OER polarization curve. c) Corrosion potential and corrosion current density and d) FE. Reproduced with permission.^[^ ^41^ ^]^ Copyright 2021, Elsevier. CoP@FeOOH catalyst for seawater splitting. (a) OER polarization curves and (b) Tafel slop values. Reproduced with permission.^[^ ^42^ ^]^ Copyright 2021, Elsevier

**Figure 6:**
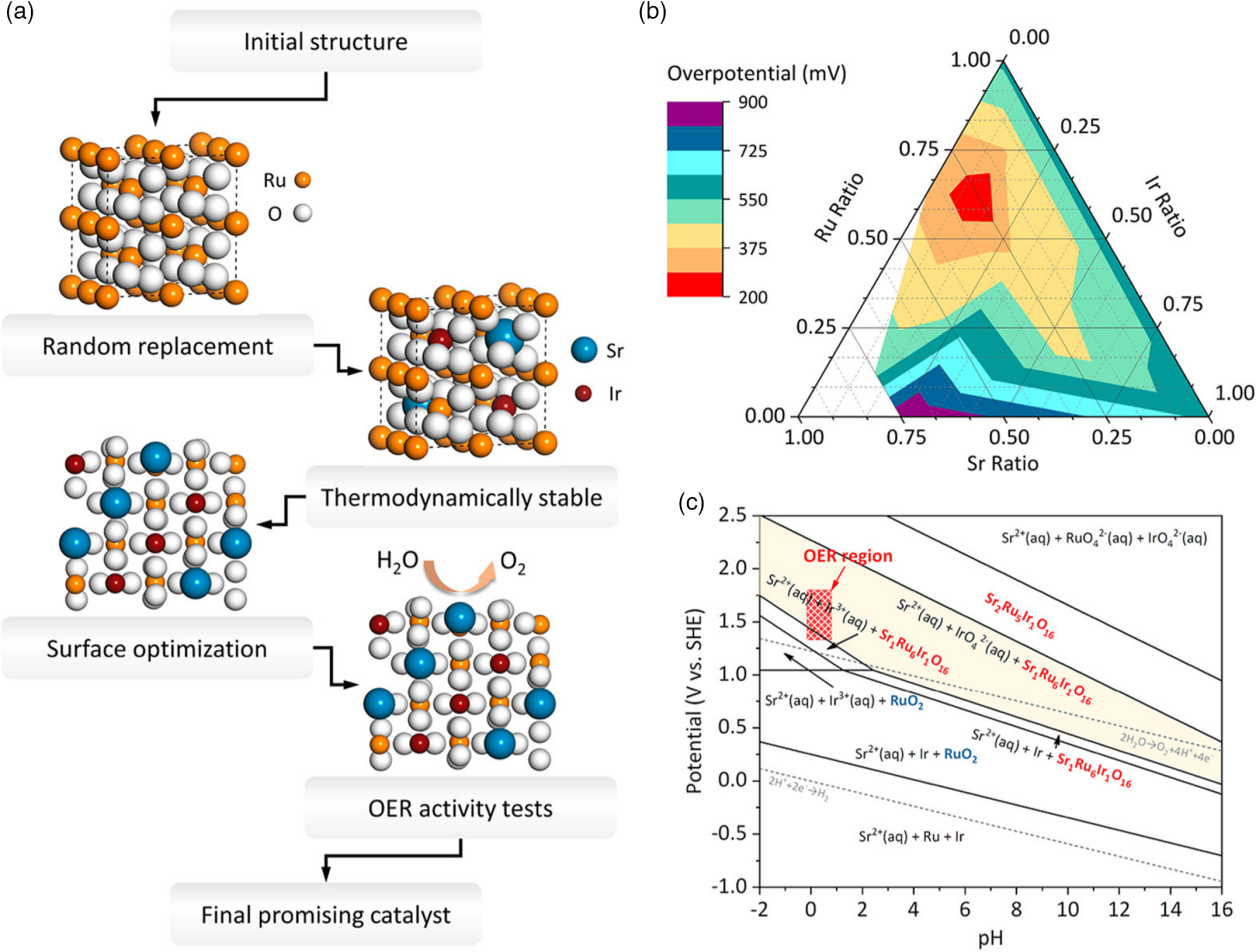
a) High‐throughput DFT simulations to establish the thermodynamically stable structure by randomly substituting the Ru with Ir or Sr. b) Different metal stoichiometry modified the OER overpotential, the red zone shows the Sr–Ir–Ru atomic ratio with the lowest overpotential. c) Pourbaix diagram of Sr_1_Ru_6_Ir_1_O_16_ and Sr_2_Ru_5_Ir_1_O_16_ oxide represents that Sr_1_Ru_6_Ir1O_16_ has been thermodynamically stable in OER region. Reproduced with permission.^[^ ^44^ ^]^ Copyright 2021, American Chemical Society.

The electronic structure defines the energy barrier for the adsorption and desorption of intermediates and substantially impacts the intrinsic reactivity of active sites. The binding free energy of selective intermediates is the key parameter to link the macroscopic properties and microscopic electronic state. An electronic modulation is a practical approach to optimizing active sites’ chemisorption energy to make them highly selective for OH^−^ intermediate. According to Norskov's theory, the anode surface provides active sites for the adsorption and desorption of intermediates associated with the metal *d*‐band center position.^[^ ^39^ ^]^ The superposition of the O 2*p* band with the metal *d*‐band favors the OH intermediates and increases the selectivity of the active center for OER. The electronic structure could be well controlled by optimizing the location, stoichiometries, and nature of dopants. In recent years, significant endeavors have been put to modify the electronic structures of active sites to accelerate their selectivity and activity for seawater electrolysis. Qiao and workers investigated the impact of Co charge state in cobalt selenide for seawater electrolysis.^[^ ^40^ ^]^ The valance state of cobalt metal in Co–Se catalyst was controlled by manipulating the mass ratio of Se powder to Co foil. The hybrid material revealed high activity, selectivity, and corrosion resistance for seawater splitting. Synchrotron‐based mechanisms were used to probe the active sites for selective OER. Results revealed that the high oxidation state of cobalt selectively binds with the O intermediate and triggers the OER process in aggressive seawater. Experimental findings revealed that the anodic performance of catalyst deteriorated by increasing the Se/Co mass ratio. The overpotential needed to deliver a geometric activity of 100 mA cm^−2^ was drastically increased from 0.28 to 0.41 V by increasing the Co: Se from 1:1 to 1:4. It has been examined that highly oxidized metal species favor OH^−^ adsorption reaction on M–O intermediate (OOH intermediate) with optimum adsorption energy and are supposed to be actual active sites. Synchrotron‐based X‐ray absorption near edge structure (XANES) also demonstrated that during electrolysis, Co^3+^ sites can be further oxidized to Co^4+^ sites, reduce the energy barrier, and oxidize the water molecule to O_2_ gas. While the 3*p* orbital of Se in higher‐Se content catalyst coordinates with oxygen 2*p* orbital, this repulsion deactivates the active centers. The electronic structure optimization to support the sequential proton‐electron transfer in the potential determining step on metal sites and electrolyte engineering increases the selectivity and activity of active sites for OER. Li et al. synthesized carbon fiber‐supported cobalt carbonate hydroxide (Co CH@CFP) and compared its selectivity and activity with Ni–Fe layered double hydroxide (LDH) for seawater oxidation.^[^ ^33^ ^]^ The Co CH@CFP exhibited high selectivity and activity for OER compared with Ni–Fe LDH. This high performance was explained by the acid–base properties of the catalyst surface determined through the pH shift method. The pH of no net charge on the material surface also called the point of zero charges reveals the surface acid–base strength for solid material and the chemisorption energy between oxygen intermediates and metal sites. The Co CH@CFP point of zero charges was found higher than the Ni–Fe LDH, imparting the lower acidity of CH@CFP and its affinity for Cl^−^ adsorption. The OH^−^ is a soft base compared with Cl^−^ and has more diffusion ability to the surface of a weak acid (CH@CFP). The electronic interaction of the electronegative metal with transition metal creates high‐valance active sites that selectively bind with the OER intermediates in the presence of Cl^−^ ions. We synthesized Au nanoclusters‐decorated gadolinium–cobalt boride nanoflakes embedded in TiO_2_ nanosheets (Au–Gd–Co_2_B@TiO_2_) that demonstrated outstanding activity (*η*@1000 mA cm^−2 ^= 510 mV) and selectivity for unpurified seawater electrolysis (FE >98% for OER and HER).^[^ ^41^ ^]^ The improved selectivity was assigned to the exposed Au–Gd–Co_2_B interface that decreased the energy barrier of the rate‐determining step from 1.17 to 0.77 eV. The highly electronegative Au with lower *d*‐band oxidizes Co to a high valance state and modulates the Co *d*‐band. Its high interaction with O 2*p* orbital stabilizes the OOH intermediates and triggers the OER over Cl^−^ oxidation (**Figure** **5**a–d). Wu et al. evaluated the electrochemical performance of core–shell CoP@ Fe–OOH structure for seawater electrolysis.^[^ ^42^ ^]^ The results suggested that instead of simple physical mixing, the strong electronic interaction between CoP and Fe–OOH revealed almost 98% FE (Figure 5e,f). The electron‐dense P atom in the CoP core modifies Fe–OOH's surface energy that selectively combines with the OER intermediates and yields high FE at a high turnover number. Cationic insertion into the lattice of 3*d* TM adjusts its orbital energy level and as a result improves the electronic interaction to optimize the binding energy (BE) of OER intermediates. Liu et al. reported the low‐crystalline, nickel foam‐supported Zr‐doped Co–Fe LDH and probed its selectivity for OER in seawater.^[^ ^43^ ^]^ It was observed that Zr^4+^ doping considerably modifies the electronic structure of LDH and exposes particular active sites for OER. High‐charge Zr coordinates with cobalt oxyhydroxide, which is believed as OER active site. Benefiting from low‐degree‐crystallinity‐optimized electronic arrangements, Zr‐doped Co–Fe LDH revealed high activity and selectivity for alkaline seawater oxidation. The high‐valance state of Ru also accelerates the OER kinetics to the back‐donation of electrons from oxygen to Ru *d*‐orbitals. Strontium electronic interaction promotes and stabilizes Ru to a high charge state that is more active and selective for seawater oxidation compared with conventional RuO_2_. It was investigated that Sr modulates the electronic structure of Ru by creating the unoccupied *e*_g_ states above the Fermi level.^[^ ^44^ ^]^ The population in *e*_g_ orbital strongly influences OER kinetics because it is directly involved in the bonding (sigma bond) with OER intermediates. The Sr incorporation in Ru and Ir oxide matrix creates more *d*‐band holes and enhances the covalency of metal *d*‐orbital with O 2*p* orbital, contributing to the high selectivity and activity in seawater oxidation (**Figure** **6**). More promisingly, the chemical doping tuned the electronic structure of transition metal and reduced the energy barrier. The electronic interaction between two or more transition metals promotes charge transfer at the interface. Recent findings demonstrated that the surface energy of Fe active sites for OER intermediates could be augmented by e^−^–e^−^ interaction between intermediates and Ni and accelerate the sluggish kinetics of OER. Fe's most stable electronic configuration is 3*d* ^5^ when Fe is in *a* + 3‐valance state, and its *t*_2g_ orbitals have unpaired electrons. The unpaired electrons coordinate with the OER intermediate through π donation. Similarly, the electronic configuration of Ni^2+^ is 3*d* ^8^ and has no unpaired electrons in π symmetry (*t*_2g_ orbitals) and causes electronic–electronic repulsion. In the Ni–Fe bimetallic system, Fe active sites’ interaction with OER intermediates can be modulated by such repulsion between nickel and intermediates, causing a remarkable reduction in the energy barrier.^[^ ^45^ ^]^

Fe is typically active in +3/+4 oxidation states; however, at high anodic potential, Fe irreversibly oxidizes to a higher charge state, dissolutes, and decreases the activity–stability factor (ASF). Fe electronic interaction with 3*d* metals and 2*p* nonmetals prevented the Fe surface from excessive irreversible oxidation. Wang et al. reported the electrochemical response of Co–Fe_2_P for seawater electrolysis. X‐rays analysis revealed that the Co surface oxidizes to a higher valance state during water oxidation, but Fe preserves its charge due to covalent interaction with P. The M─P bond creates oxide/hydroxide active layers on the Co–Fe surface and optimizes the Fe active sites for OER intermediates.^[^ ^46^ ^]^ In multimetal catalysts, the stoichiometry of each metal has a remarkable influence on the electronic structure of hybrid material. Ni sites are considered more selective for OER in Ni–Co bimetallic catalyst because its 3*d* band has a higher superposition with *p*‐orbital. A recent report shows that a higher Ni/Co ratio has more activity and selectivity for seawater oxidation.^[^ ^47^ ^]^ Song et al. reported that sodium cobalt−iron pyrophosphate (Na_2_Co_1−*x*_Fe_*x*_P_2_O_7_/C) was fabricated on carbon cloth and revealed good selectivity for seawater oxidation.^[^ ^48^ ^]^ At high anodic potential, surface reconstruction of the catalyst from pyrophosphate to oxyhydroxide was observed, which is responsible for increased efficiency. The electrochemical modulation triggers the Co oxidation to a high valance state with P and Na loss, and these electrophilic active sites promote seawater adsorption. The binding affinity of active sites for OER intermediate increases with Fe concentration, and strong electronic interaction ensures Fe's periodic redox behavior in seawater oxidation.

This is a well‐known fact that Fe impurities substantially enhanced the OER kinetics. However, it is still debatable whether Ni or Fe is the actual active center for OER intermediates. Chang et al. developed P‐ and Fe‐doped Ni–Se_2_ nanoporous film and evaluated its activity for seawater electrolysis.^[^ ^49^ ^]^ The results revealed that cation Fe electronic interaction with Ni–Se_2_ film improved seawater oxidation's selectivity and FE. The P atom insertion in bimetal lattice facilitates the charge transfer, prevents the dissolution, and improves the ASF. Computational analyses were performed to identify the active sites for OER intermediate by considering the Fe site, Ni site, and bridging Se center on the Fe–NiSe_2_‐exposed surface. The results demonstrated that the limiting potential of OER on Ni sites was much lower than the limiting potential on Fe and Se sites. This lower limiting potential and other experimental results demonstrated that Ni is the active site for OER intermediate in this hybrid material. The limiting potential of OER on partially oxidized FeO–NiSe_2_ was calculated, and surprisingly, it was almost similar to the unoxidized surface that revealed that surface oxidation has a negligible impact on the reactivity of active sites. The free energy profile for each coordinate demonstrated that Fe–NiSe_2_ has a much lower limiting potential than Ni–Se_2_, suggesting the unbeatable role of electronic interaction. It was also inspected that Ni atom metallically bounded with Fe atom and has much faster kinetics for water oxidation. The P atom interaction with TM increased the density of states on the Fermi level and improved the electronic conduction. The electronic interaction changed the bond structure of metals and was considered an effective strategy to destabilize the water molecule on the active center. This destabilization increases water's charge polarizability and decreases the activation barrier needed to break the O─H bond. For example, electrochemical deposition of Pt and its electronic interaction with IrO_2_ alters the Ir─O bond length and regulates the electronic distribution on the exposed center. The charge migration from HOMO of higher‐energy orbitals to the LUMO of the electronegative atom provides abundant selective sites for OOH stabilization.^[^ ^50^ ^]^

**Figure 7:**
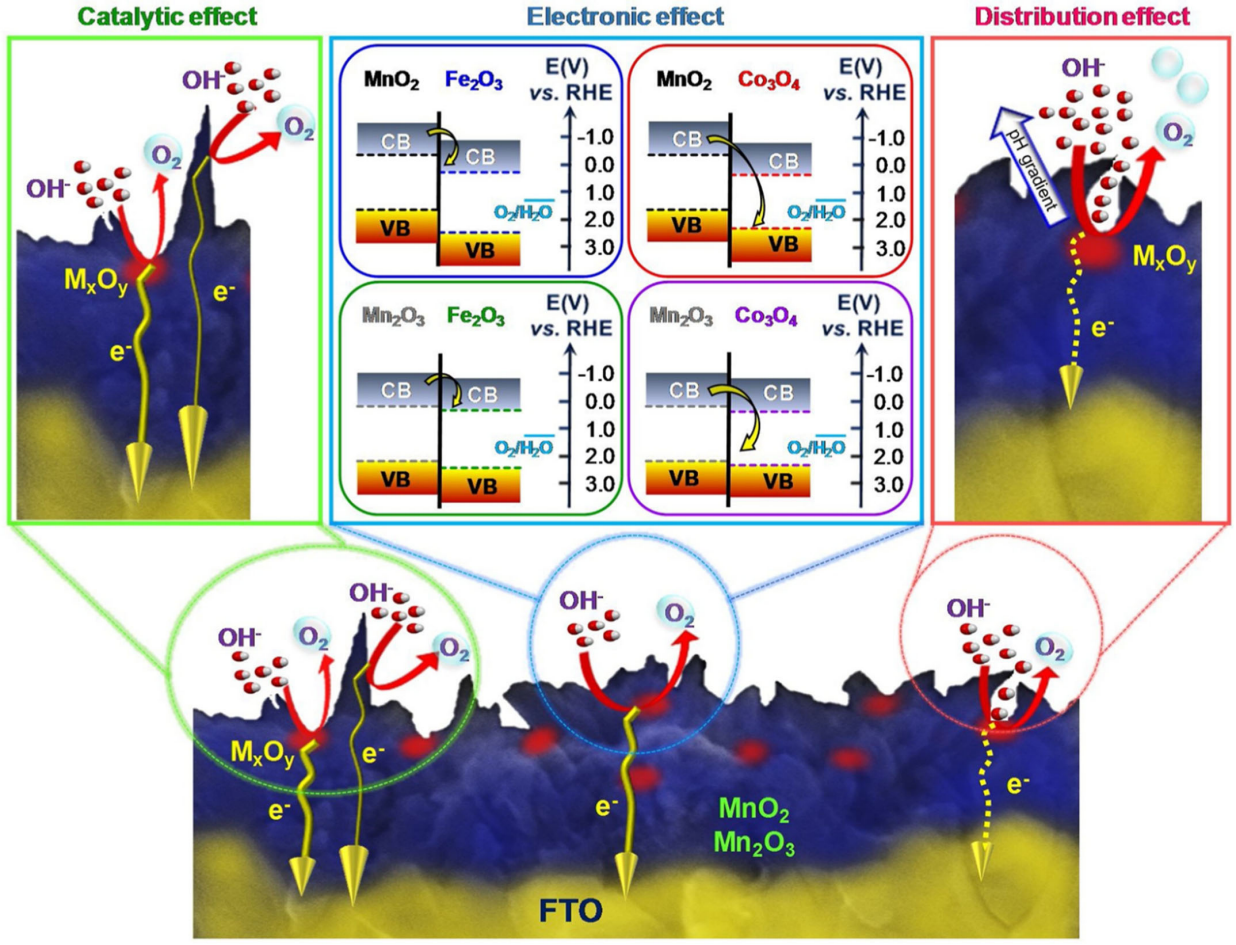
Sketch representing the different OER efficiency of Mn_2_,O_3,_ and MnO_2_ electrodes. M_*x*_–O_*y*_ = Co_3_O_4_, Fe_2_O_3_.The middle panel comprises a schematic energy band diagram of Co_3_O_4_–Mn_2_O_3_, Co_3_O_4_–MnO_2_, Fe_2_O_3_–Mn_2_O_3_, Fe_2_O_3_–MnO_2_, and systems, with estimated energy levels concerning the RHE) scale, CB represents the conduction band while VB represents the valance band. Reproduced with permission.^[^ ^51^ ^]^ Copyright 2020, Elsevier.

Surface reconstruction is also an effective strategy for electronic and morphological alteration in metal oxide, modifying the metal charge state and creating surface defects needed for aggressive seawater electrolysis. Maccato and coworkers synthesized Mn_2_O_3_, and MnO_2_, functionalized with Co_3_O_4_ and Fe_2_O_3_ by radio frequency (RF) plasma‐assisted process and probed its efficiency for seawater electrolysis.^[^ ^51^ ^]^ Among the different anodes, the Co_3_O_4_–MnO_2_ composite demonstrated faster kinetics for OER without Cl interruption. Experimental and theoretical routes were used to answer the question, “why does Co_3_O_4_ functionalization yield better performance?” **Figure** **7** shows the simultaneous contribution of in‐depth spatial distribution, electronic, and catalytic effects. The arrow thickness in the catalytic effect square corresponds to the current density originated at particular reaction sites. Figure 7 reveals that the number of electrons produced by the composite surface is much higher than these generated by individual component. The coexistence of the heterojunction between functionalizing agent (Fe_2_O_3_, Co_3_O_4_) and Mn‐based oxide (MnO_2_ and Mn_2_O_3_) further synchronized the material activity for OER due to better charge separation (Figure 7). At p–n heterojunction, Co_3_O_4_ receives the charge density from n‐type Mn oxide semiconductor while in n–n heterojunction electron moves to the lower energy band of Fe_2_O_3_ from the higher energy conduction band of MnO_2_ or Mn_2_O_3_. The energy difference between the Co_3_O_4_ valance band and Mn–O conduction band is higher than the Fe_2_O_4_/Mn–O and is supposed increase charge separation. The results further supported that the energy gap is wide for MnO_2_ (energy position vs. normal hydrogen electrode (NHE), valence band [VB] = 1.7 eV, CB = −0.3 eV)‐based composite compared with Mn_2_O_3_ (energy position vs. NHE, VB = 2.2 eV, CB = 0.2 eV) and has faster kinetics. The difference in depth distribution analysis reveals that Co_3_O_4_ has more contact near the surface region and increases the heterojunction number.

Jiang et al. investigated the impact of different dopants on the catalytic activity of manganese oxide for seawater oxidation.^[^ ^52^ ^]^ The results revealed that Mn–O chemically transformed to other oxides, facilitating the reaction kinetics at the electrode junction. The Fe doping and its electronic interaction significantly enhanced the Mn–O reactivity for the OER intermediate. The Fe interaction stretched the Mn─O bond, decreased the O─H bond energy, and increased the rate of O─O bond formation. The orbital configuration revealed hat Mn has five electrons in 3*d* orbital while Fe has six electrons. Fe atom doping substituted the Mn atom, leaving behind the sixth electron loosely bound to the Fe atom, participating in bond formation, decreasing Mn─O bond energy, and improving OER kinetics. It has been revealed that vanadium doping creates a vacancy in manganese oxide structure and improves active sites’ hydrophilicity, activating the OH^−^ ions. The experimental results endorsed that both Fe and V doping significantly boost the OER efficiency as its electronic interaction alters the surface electronic structure.

### 4.2 Oxygen Vacancies

The presence of oxygen vacancies on the catalyst‐exposed surface constructively modulates the electronic structure, surface features, alters the interaction affinity of electrode surface for electrolyte, and thus tunes the active sites for OER intermediates. The required energy for surface oxygen vacancies is a crucial factor in modulating the catalytic activity of metal oxides. Shimizu and co‐workers quantify the energy for the formation of surface oxygen vacancies for different oxides (*Eo*_vac_).^[^ ^53^ ^]^ Theoretical calculation demonstrated that electron affinity, bandgap, and bulk formation are the factors that regulate the *Eo*_vac_. Electrons are conducting in the defect states available in the valance band, conduction band, and Fermi level after surface oxygen vacancies. The intermediates preferably adsorb on the defect sites, and its adsorption energy depends on the *Eo*_vac_.

**Figure 8:**
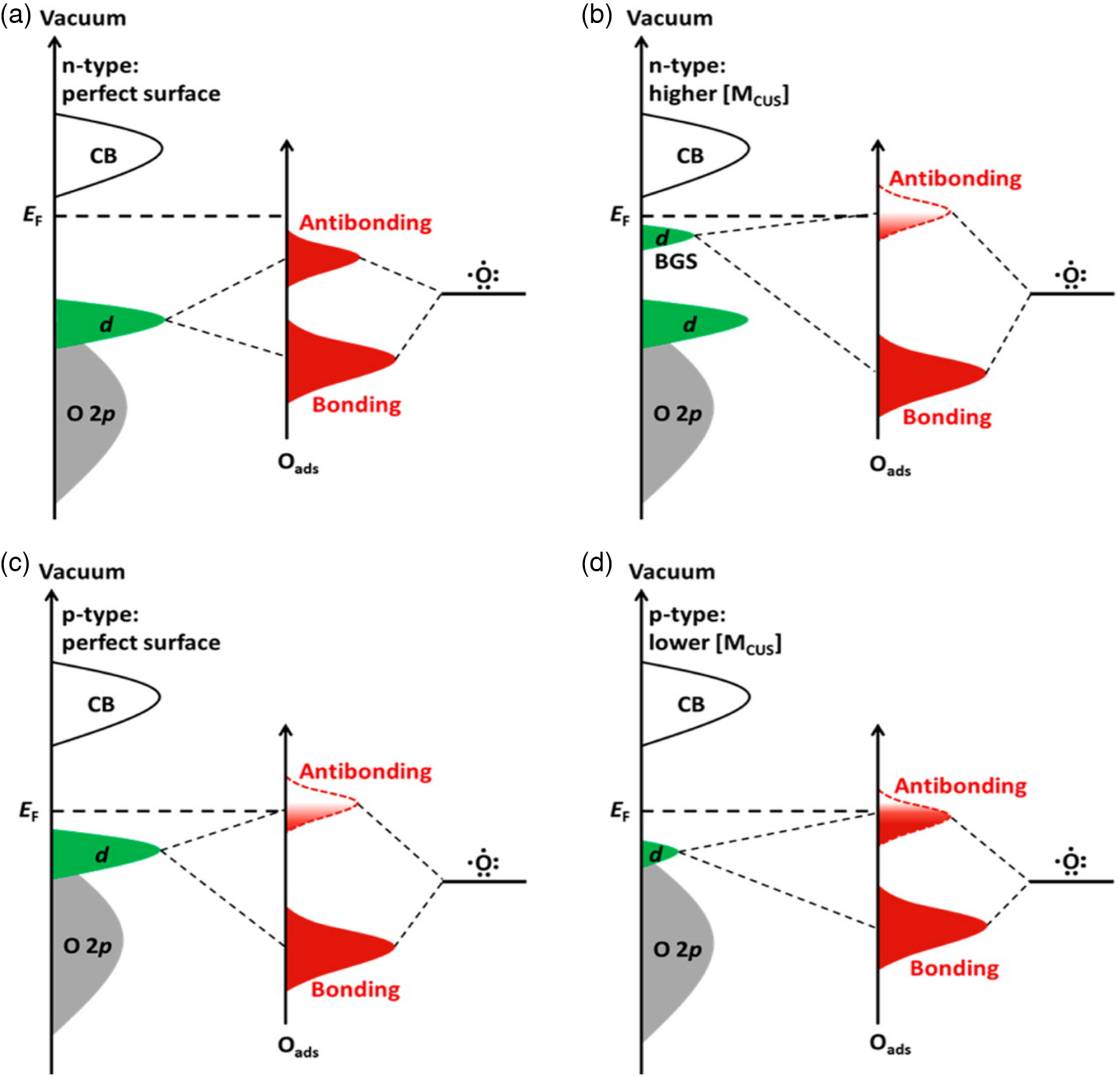
Systematic representation of O 2*p* orbital bond formation with the highest occupied *d*‐state of TMO. The VB of the most stable insulators or semiconductor TMO comprises the *d*‐state of TM cation and the O 2*p* state. a,b) *n*‐type TMO bonding with O 2*p*, where highest occupied filled state at lower energy relative to Fermi level and antibonding states are usually filled. The surface oxygen vacancies create a bandgap caused by unpaired *d*‐electrons and lead to stronger adsorption of O due to uplift of *d*‐TMO antibonding states relative to Fermi level. c,d) *p*‐type TMO bonding with O 2*p* where highest occupied states are much closer to Fermi level, and antibonding states are less populated than *n*‐type TMO─decreasing the highest occupied states relative to Fermi level, causing the more filling of antibonding states and decreasing the interaction. The difference between the highest occupied state and Fermi level defines the electronic origin and surface reactivity of TMO. Reproduced with permission.^[^ ^55^ ^]^ Copyright 2016, American Chemical Society.

The formation of oxygen vacancies modulates the electronic structure, creates a new active phase, and increases the charge and mass transportation at the interface (electronic and ionic conductivity). These unique features substantially impact the adsorption–desorption process and considerably reduce the potential barrier for O–H intermediates. The oxygen density is improved with the energy difference between the Fermi level and O 2*p* band and directly impacts the M─O bond.^[^ ^54^ ^]^ Bin Liu and co‐workers proposed the model based on band structure and molecular orbital theory to understand the impact of oxygen vacancies on electronic modulation.^[^ ^55^ ^]^ **Figure** **8** demonstrates the bond formation of atomic oxygen on the *d*‐metal surface. Atomic oxygen accepts the electronic density from metal cation during bonding due to its low orbital energy. Coordinatively unsaturated metals oxide with 3*d* or 4*d* valance electrons provides active sites for intermediate adsorption. OER intermediates’ interaction with metal was attributed to O 2*p* coupling with the highest occupied metal *d*‐states. As the bonding states are occupied, they have lower energy than the fermi level and are located far below, while the position and population of antibonding states depend on the relative energy of Fermi level and occupied *d*‐states. Intermediates are adsorbed strongly due to unfilled antibonding states if the occupied *d*‐states are at higher energy than the Fermi level. The authors explained the model for three different categories, that is, metallic transition metal oxide (TMO), p‐type, and n‐type TOM. The intermediates’ adsorption is too weak on n‐type TMO as the antibonding orbitals are filled due to the low energy of occupied states. The creation of oxygen vacancies creates unfilled states and pushes the antibonding to a higher energy than the Fermi level. The introduction of this bandgap increases the reactivity of active sites for OER intermediate. In contrast, the occupied states in controlled p‐type TMO are close to the Fermi level and partially filled antibonding state. These partially filled states in p‐type TMO (e.g., NiO, MnO_2_) have stronger interaction with OER intermediate and reveal higher oxidation activity than n‐type TMO (e.g., TiO_2_). The d band theory also successfully explained the difference between surface reactivity of TM and TMO. The reaction intermediate interacts with both the *s*‐ and *d*‐states of the metal center. In contrast, intermediate only reacts with the *d* state on the TMO surface, so TMO shows less surface reactivity and works well in seawater electrolysis. Oxygen vacancies enhance the interaction affinity of anode surface for OH^−^ groups at anode bias and decrease the adsorption capacity of active sites for OH^*^ intermediate that facilitates the O─O bond formation from the electrolyte route. Wang et al. reported that oxygen vacancies through S doping modify the electronic density, increase the defective active sites that are selectively bound with the OER intermediates, and make the dissociation of O─H bond thermodynamically favorable at low cell voltage.^[^ ^38^ ^]^ Oxygen vacancies in the vicinity of Ni/Co increase the polarizability of the H─OH bond and improve the OER kinetics. The recent finding demonstrates that S doping in CuO structure increases the electrical conductivity and proton‐electron transfer rate. From the density functional theory (DFT) calculation, Wang et al. found that synergy of oxygen vacancies with the selective metal of the appropriate occupied state boosts the efficiency of OER. Theoretical measurements were performed for nine different models by considering the O─O bond formation through the electrolyte route (M–OH, M–O, and M–OOH) and screened the intermediate adsorption process. The geometries were optimized by considering the defect‐free and defect‐rich models. The adsorption energy of O intermediate on the surface of an ideal catalyst was used for comparison. Results unveiled that sulfur vacancies and Ni insertion in the geometry of the regular structure improve the OER performance. The OH* intermediate adsorbed too strongly on the Cu–S site, hindering the electron removal process (from OH^*^ to O^*^). The free energy profile has shown that vacancies’ creation optimized the bonding strength of OH* with active sites. The generation of vacancies decreases the binding strength while the neighboring Ni sites promote the O─O bond formation due to the strong hydrogen BE effect. The defects affected the adjacent Cu atoms, reduced the energy gap between 3*d* orbital and Fermi levels, and boosted the reaction kinetics. The oxygen vacancies through electronegative further narrow this gap and increase the reaction rate.

**Figure 9:**
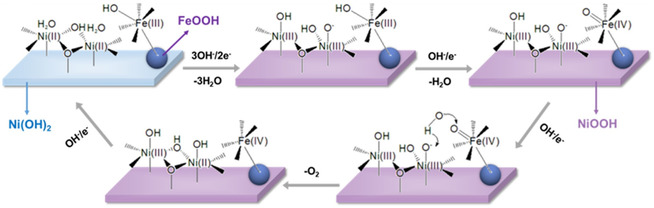
OER mechanism on the surface of Mo–Ni foam‐supported, S‐doped Ni–Fe LDH. Reproduced with permission.^[^ ^56^ ^]^ Copyright 2021, Elsevier.

The oxygen vacancies through anion doping increase the bond polarizability, stabilize the OER intermediate, and enhance the desorption rate. **Figure** **9** shows the impact of S doping to regulate the electronic structure, decreasing the free energy and improving the bonding between OER intermediates and active sites reported by Chai and co‐workers. The results revealed that Fe oxidized to Fe–OOH at anodic potential, covalently bound with Ni(OH)_2_. The oxidation reaction generates Ni^3+^ dimer with the elimination of protons from OH or water molecules. Thnext step is the preequilibrium step comprising a proton‐coupled electron transfer mechanism at Fe ^3+^ sites that eventually oxidizes to Fe^4+^═O. The third step involved the reaction of Fe^4+^═O sites with Ni^3+^–O and OH that produced Fe^2+^–Ni^2+^–OH sites. In a final step, the reaction sites are regenerated and exposed for cyclic response. The mechanistic studies demonstrated that electronegative S coordinates with hydroxyl anion give *O*–H…S and this H bond is stronger than O–H.O (Figure 9).^[^ ^56^ ^]^ This interaction shifts the electronic density from S to H atoms, creates abundant electrophilic active sites, and facilitates OH^−^ on MO^*^, making this process thermodynamically feasible. Although heteroatom doping is an effective strategy for vacancy creation, leaching from the electrode surface at the high anodic potential in aggressive seawater is the bottleneck. For example, Gupta et al. synthesized cobalt iron oxy boride with flower‐like morphology containing boron shells around Co_3_O_4_ small particles.^[^ ^57^ ^]^ The boronization process chemically reduces the catalyst, creates the oxygen vacancies, and improves the charge transfer rate and selectivity of the anode for OER. The improved activity was attributed to the active Fe sites, oxygen vacancies, and the formation of selective Co–OOH phase on the surface of the catalyst that promotes O─O bond formation. However, a considerable reduction in FE from 100 to 74% was observed at high current density, indicating Cl^−^ interference. The electrolyte analysis after the water oxidation experiment reveals substantial leaching of B.

Recent findings reveal that electronegative atom coordination with dense positive charge defects created by the anodization process has the potential to inherently bind these atoms in structure lattice. We developed amorphous S–Cu_2_O–CuO nanoneedles for natural seawater electrolysis.^[^ ^58^ ^]^ The Cu_2_O–CuO nanoneedles were directly grown on the Cu substrate in an alkaline electrolyte (with 0.1 m NaBH_4_), creating plentiful oxygen vacancies and strong metal–support interaction with a wider energy gap that accelerated the charge and mass transport at the interface. S incorporation in the structure through anodization approach creates a firm bond between S and Cu in the designed structure, increases the oxygen vacancies, and disorders the structure. We have analyzed the morphology and elemental composition of the catalyst before and after its use for water electrolysis for comparison. Notably, no change was observed in the S content after stability tests in aggressive seawater electrolyte, suggesting that the promotional effect of sulfur remains strong throughout the process. The results indicated the adsorption of oxygen functionalities or surface oxidation during water oxidation reaction very commonly observed in heteroatom‐doped transition metals. It was anticipated that the S–Cu^2+/3+^ redox couple might be the actual active phase to govern OER that ensures the metal redox couple's periodicity. For further justification, we also collected the inductively coupled plasma–optical emission spectroscopic (ICP–OES) measurement of KOH electrolyte after 20 h of continuous electrolysis. The results demonstrated the sulfur‐free electrolyte, suggesting that sulfur species retain their inherent strong interaction with the metal in the composite and act as active phase, rather than being dissolved in the electrolyte, as usually observed in the nonoxide‐based catalyst.^[^ ^59^, ^60^ ^]^ The recent findings suggested that oxygen‐deficient surface oxides (Cu–O_*x*−δ_) formed in situ through electrochemical oxidation effectually catalyze the electrolysis process. However, the promotional effect of S remaining in the oxidized catalyst is equally effective by assisting in situ‐formed metal oxo‐/hydroxide OER active sites on the surface of S‐doped CuO.^[^ ^58^ ^]^ It is also observed that oxygen vacancies promote the OER kinetics and selectivity by forming O─O bond through two MO^*^(M represents active sites) intermediates instead of the general peroxide route. Abe et al. found that oxygen vacancies in the vicinity of Mn oxide increase the selectivity for OER, and its magnitude has a linear relation with the density of vacancies.^[^ ^61^ ^]^ They synthesized fluorine doped tin oxide (FTO)‐supported Mn–O layer by electrodeposition of layer Na–MnO_2_. The oxygen vacancies were created by thermal treatment and their activity for simulated seawater oxidation was evaluated. The X‐ray investigation revealed that the onset point for oxygen vacancies was 200 °C and decreased Mn–O's charge state. The disorders in the surface structure were found at higher temperatures (400 °C), increasing the oxygen vacancies and further enhancing the OER selectivity up to 87%. This higher selectivity was attributed to the presence of oxygen vacancies that trigger the O─O bond formation through lattice oxygen route instead of general OOH route.

The oxygen vacancies with multivalance metals increase the polarizability, reduce the bond strength and energy barrier, and increase the OER kinetics and selectivity. Ramani and co‐workers investigated that Pd interaction with ruthenium oxide creates the oxygen vacancies and stabilizes Ru in two different valance states.^[^ ^34^ ^]^ Pb_2_Ru_2_O_7_ comprises a high concentration of Ru^5+^ in addition to Ru^4+^ and reveals high activity and selectivity for seawater electrolysis. This higher activity was attributed to the high‐valance Ru and oxygen vacancies, providing active sites to OER intermediates with optimum adsorption energy. The oxygen vacancies promote the O─O bond formation through the lattice oxygen route as stated above and increase the selectivity of hybrid material for seawater oxidation.

The ex situ‐oxidized TM also exhibits a strong tendency to stabilize OER intermediates in alkaline seawater. Acid‐corroded Ni–Fe LDH has been reported for alkaline seawater where hydrochloric acid (HCl) was used to erode the surface structure, resulting in exposures of high valance active sites that can be readily transformed to MOOH, decreasing the kinetic barrier for O─O bond formation.^[^ ^62^ ^]^

### 4.3 Passive Layer

Even with a highly efficient anode material and electrolyte engineering, the aggressive Cl^−^ anions in seawater at higher concentrations can corrode catalyst surface through the M–Cl–OH formation mechanism. This corrosion collapses the substrate skeleton, blocks the active sites, impedes reaction kinetics, and reduces product purity. In recent studies, researchers used a protective layer next to the OER actual active sites to increase the selectivity and durability of anode material for seawater oxidation. Koper and co‐workers reported that Mn–O thin layer coating on the IrO_2_ catalyst drastically reduces the IrO_2_ selectivity for CER from 86% to 7%, making it a selective site for OER intermediates.^[^ ^63^ ^]^ Mn–O coating disfavors the catalyst surface's Cl^−^ diffusion and adsorption and substantially enhances the anode durability in aggressive seawater. However, this type of coating masks the OER active sites and decreases the geometric activity and as a consequence considerably reduces the overall efficiency. In addition, all metals can act as OER active sites at high anodic potential and catalyze the OER and Cl^−^ oxidation simultaneously. Dai and co‐workers demonstrated that when polyatomic anion layer is integrated on the surface of catalyst or buried under the active layer, it increases the corrosion resistance of active sites. This observation is attributed to its cation selectivity which promotes the H^+^ ions to diffuse into bulk and inhibits the Cl^−^ anions from interaction with the anode.^[^ ^37^ ^]^ They developed a multilayer anode comprising Ni–Fe LDH homogeneously coated on sulfur‐modified Ni foam. Anodization approaches were used to functionalize the multilayer with negatively charged polyanions, resulting from the underlying NiS layer. This anionic passive layer repelled the Cl^−^ anions in seawater and is responsible for the high corrosion resistance. It was found that bare Ni foam and NiS retained FE of less than 35% and lasted for only 20 min. The Ni–Fe LDH exponentially enhanced the FE of anodic reaction up to 99.9%, and now it has sustained a high current density for more than 1000 h. The results that Ni–Fe LDH without Ni–S layer shows stability only for 12 h endorsed the role of hybrid material. The superior strength and selectivity were attributed to the carbonate and sulfat‐ inserted Ni–Fe LDH and the Ni–S anion layer.

**Figure 10:**
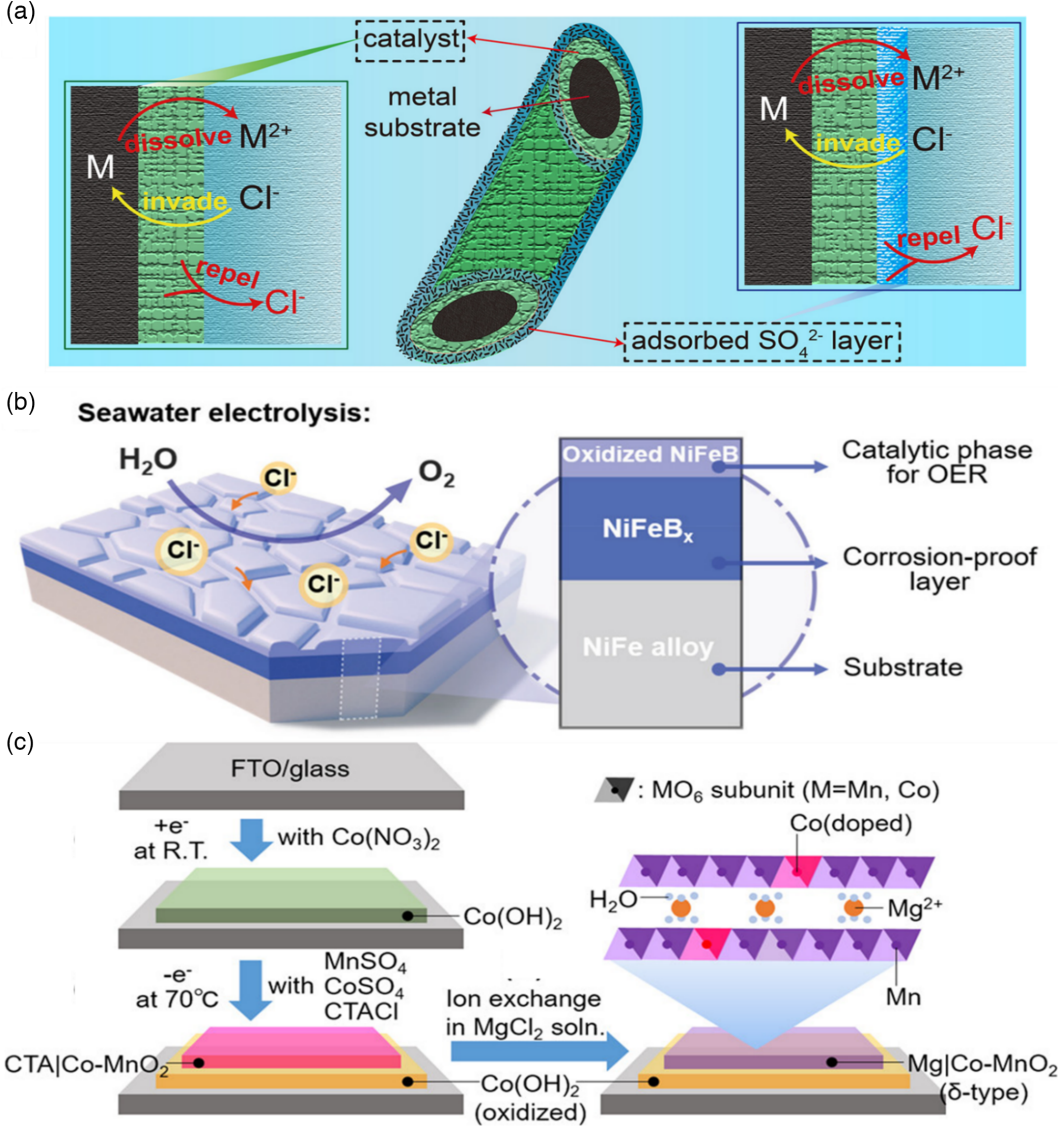
a) SO_4_^2−^ protecting layer has been fabricated to protect the metal substrate from chloride corrosion in seawater electrolysis. Reproduced with permission.^[^ ^64^ ^]^ Copyright 2021, Wiley‐VCH. b) Illustration of the multilayered anode composed of the Ni–Fe alloys substrate for structural support, NiFe–B alloys as a corrosion‐proof layer, and surface‐oxidized Ni–Fe–B as OER active layer shows higher selectivity and corrosion resistance in seawater electrolysis. Reproduced with permission.^[^ ^32^ ^]^ Copyright 2021, Wiley‐VCH. c) Flow diagram representing the construction of bilayer structure comprising Mg‐inserted, MnO_2_‐doped Co (upper) and Co (OH)_2_ (under). The steps are electrodeposition of Co (OH)_2_ under layer and electrodeposition of cetyltrimethylammonium Co–MnO_2_ followed by ion exchange process for Mg intercalation. Reproduced with permission.^[^ ^66^ ^]^ Copyright 2020, American Chemical Society.

Ma et al. found that sulfate anions additive in the electrolyte with optimum concentration drastically improves the selective and corrosion resistance of anode material in seawater electrolysis.^[^ ^64^ ^]^ Both in situ experimental and theoretical insights revealed that SO_4_^2−^ additive in electrolyte could selectively be adsorbed on the anode and alleviate the corrosion resistance by repelling the Cl^−^ anions through electrostatic repulsion (**Figure** **10**a). The results revealed that this electrostatic repulsion is extendable to other mono‐ and multimetal catalysts. The classical molecular dynamics simulations unveiled that anodic potential accelerates sulfate anion movements toward the anode. In the absence of sulfate additives, Cl^−^ ions diffused and adsorbed on the anode surface. The sulfate anions have higher mass diffusion (1.070 × 10^−9^ m^2^ s^−1^) than that of Cl^−^ (2.030 × 10^−9^ m^2^ s^−1^) and are preferably adsorbed on the anode surface. The electrostatic repulsion pushes the Cl^−^ away from the anode surface and increases the corrosion resistance. However, the presence of this anion layer on the anode surface affected the OER kinetics as it impedes the OH^−^ attack and increases the potential barrier for OOH formation. Therefore, optimum stoichiometry of these anions and their isotropic engineering are needed to further improve the ASF. In comparison with simple TM‐based catalyst, anion‐incorporated anode material oxidizes the metal to a higher valance state. However, anion presence increases the activity of active sites, generates some new selective phases, and increases the corrosion resistance of anode via electrostatic repulsive forces. 3D heterolateral Ni_3_S_2_/Co_3_S_4_ (Ni–Co–S) nanosheets were fabricated on Ni foam and show excellent OER selectivity and corrosion resistance.^[^ ^65^ ^]^ It was observed that the M─S bond disappears during OER and hydroxide layer shell the metal core, impedes the Cl^−^ diffusion, and promotes the O─O bond formation at low overpotential. The in situ‐generated core–shell feature and S anion collectively ensure the firm adhesion of active metal oxy/hydroxide sites with the current collector. As a result, both factors increase the charge transfer resistance, optimize the OER intermediate adsorption, and alleviate the Cl^−^ corrosion.

Li et al. developed a MoS_2_ layer to trigger the OER and HER kinetics in simulated seawater and coated both sides with the Ni_3_S_2_ layer to increase the corrosion resistance of active sites.^[^ ^67^ ^]^ Polyatomic anion‐rich Ni–S layer shields the Mo–S layer from Cl^−^ adsorption, and hybrid material reveals sustained behavior in harsh conditions. Although at high anodic potential Mo 3*d* has an affinity for Cl^−^ anions, the polyatomic anion layer does not permit the existence of these naughty anions and increases the corrosion resistance and selectivity of anode materials. The Ni_3_S_2_ layer provides adsorption sites, and its interaction with the Mo–S layer increases the activity of active sites. The sandwich sulfide was found to be oxidized to sulfate, which repels the Cl^−^ anions from the OER active sites.

It has been reported that nonmetal interaction with cation metal regulates the adsorption energy of active metal sites and increases the electronic conductivity of hybrid material. Other benefits include oxidizing to active N–O phase (*N* = nonmetals, e.g., P, S, B), serving as a passivation layer, preventing the metal dissolution, and increasing the activity–stability factor. Chang et al. synthesized Fe‐ and P‐doped NiSe_2_ electrodes that perform well in aggressive natural seawater electrolysis.^[^ ^49^ ^]^ The insightful investigation revealed that P anion concentration up to a certain level decreases the charge transfer resistance, creates a P–O passive layer that prevents the Se dissolution, and increases the selectivity of anode material for OER. Transition metal phosphides are only dissolved in high oxidizing acids, for example, HNO_3_, and studies revealed that P content directly relates to corrosion resistance. The P content can be tuned by controlling the reaction parameters. The metal phosphide has three unique features responsible for high corrosion resistance. 1) Metal dissolution rate decreases when electronically interacting with P. 2) P contents change the surface structure of catalyst with high immunity against Cl^−^ attack. 3) Metal phosphide can be chemically oxidized to phosphate, shielding the metal core from Cl^−^ attack and having less solubility in water than other ionic species.

The various mechanisms have been demonstrated to explain the reason of anticorrosive nature of metal phosphorus.

The phosphate layer acts as a diffusion barrier and reduces metal dissolution. Hypophosphate can be adsorbed at an electrode–electrolyte junction and acts as a diffusion barrier. Phosphorus with the electron‐dense cloud is formed when covalently linked with metals and prevents the Cl^−^ attack.^[^ ^68^ ^]^ The M─P bond elongation increases the water oxidation kinetics at high anodic potential due to the quick one‐proton‐one‐electron equilibrium between M^3+^–OH and M^4+^–O, while phosphate ions accept the soft proton and decrease the activation barrier for O–O coupling.^[^ ^69^ ^]^ Wu et al. synthesized the core–shell structure of CoP@ Fe–OOH and found it highly stable in aggressive seawater electrolysis.^[^ ^42^ ^]^ After long‐term immersion experiments, no corrosion pits and structure degradation were observed on the catalyst's surface, which revealed the corrosion resistance and structural stability of the catalyst in actual seawater. The results showed that the CoP core repels the Cl^−^ anions due to its high surface negative zeta potential. The pristine CoP has high corrosion potential and lowers the corrosion current density. These gains are attributed to its electronic interaction with electrophilic Fe–OOH active sites, which slightly decreases the corrosion resistance of the structure. The electronic exchange between atoms of P with Co and Fe increases the thermodynamic stability of the system, reduces metal dissolution, and enhances corrosion resistance and chemical stability. The results revealed that P interaction with Co exposes more active–passive layers and repels Cl^−^ ions more effectively due to optimum band overlap between Co 3*d* and P 2*p* than Fe 3*d*.

It has been investigated that this heteroatom doping creates the passive layer, repels the Cl^−^ ions, and reduces the thermodynamic favorability of metal dissolution. As a result, heteroatom doping increases the corrosion resistance and mechanical stability of electrode material direly needed in seawater electrolysis. The heteroatoms‐doped TM surface oxidizes to the corresponding oxide/hydroxide, provides active sites with optimum adsorption–desorption energy, triggers the OER kinetics and inner oxidized heteroatom core, and increases the electronic conductivity, charge transfer coefficient, mechanical stability, and Cl− corrosion resistance.^[^ ^70^ ^]^ As discussed earlier, the ideal situation for seawater oxidation is to broaden the OER potential range as much as possible, engineering the active sites that selectively promote the OER and impede the CER. The corrosion resistance of anode material further increases the practical viability of the sustainable electrolysis process. Li et al. reported multilayered anode to fulfill the multiple demands of seawater oxidation. The theoretical calculations revealed that simple Ni–OOH has low affinity for both OER and Cl intermediates and impedes the kinetics of both OER and CER.^[^ ^32^ ^]^ It has been observed that Fe doping substantially improved the reaction kinetics for both OER and CER. Hence to increase the reactive sites selective for OER intermediate, a passive layer should be created to mask the active sites from Cl adsorption. Multilayered electrodes consist of Ni–Fe substate as structural support to avoid sintering, Ni–Fe–B_*x*_ interlayer as a corrosion‐proof layer, and topmost layer of Ni–Fe–B_*x*_ as particular sites for OER (Figure 10b). The experimental findings suggested that multilayer drastically improves the corrosion resistance of the material. Theoretical calculations demonstrated that the outermost B layer is present in metaborate, increasing the material's corrosion resistance and creating and stabilizing the catalytic actual active phase. The DFT calculations unveiled that metaborate species optimized the active site's adsorption energy, reduced the energy barrier, and stabilized the OOH intermediate. This electronic optimization is that electronic density shifted from B to Ni atoms and Fe atoms, and Fe also received electronic density from Ni, creating electrophilic sites that facilitate the oxidation process. It has also been inspected that metaborate species increases the covalency of the metal–OER intermediate bond by increasing the bond length and optimizing the surface adsorption capacity of active sites for OER intermediate. Gupta et al. synthesized Co–Fe–O–B and investigated the metals and B dissolution rate in alkaline and neutral seawater.^[^ ^57^ ^]^ The postexperiment electrolyte analysis revealed that the B shell is stable in alkaline seawater but leaches out from the metal surface in neutral seawater. The significantly low FE, leaching of B, and structure disorders in neutral conditions endorsed that electrolyte engineering has a vital role in the anticorrosive layer's proper functioning. Incorporating a controlled amount of more than one nonmetal has a more favorable impact on the activity and stability of active sites for seawater electrolysis, revealed by Wahab and coworkers.^[^ ^71^ ^]^ The S and B doping in Co–Fe hydroxide lattice increased the bulk conductivity, decreased the charge transfer resistance, and optimized the surface energy for OER intermediates. The results have shown that dual doping increases oxygen vacancies, reduces the energy separation between O 2*p* and metal 3*d* center, and improves the electronic conduction, charge transfer rate, and corrosion resistance. The electronic reshuffling between metal–nonmetals and oxidized anionic layer (SO_*x*_− and BO_*x*_−) enhanced the hydrophilicity–aerophobicity and shielded the anode surface from Cl^−^ corrosion. Although this negative layer prevents Cl^−^ diffusion and adsorption, it also hampers the nucleophilic attack of OH^−^ anions. It has been found that electrophilic metals with a high valance state, for example, Cr doping, facilitate the OOH bond formation and increase the turnover frequency (TOF). Cr's highly oxidized valent state triggers the (Cr^δ+^(OOH)^δ−^) structure, increases the water feed flux, and further improves the shielding of active sites from the anion.

It is highly demandable to develop an efficient passive layer that concurrently increases the corrosion resistance and enhances active sites’ intrinsic activity by optimizing the electronic structure of the catalyst. The optimum metal–nonmetal bond can polarize the water molecule and increase the rate of O─H bond dissociation.

The Cl^−^ oxidation thermodynamics can be controlled using the current collector with high resistance. Juodkazyte et al., reported that NiO‐loaded fluorinated tin oxide (FTO) oxidizes water molecules through lattice oxygen evolution mechanism.^[^ ^72^ ^]^ At high anodic potential, OH^−^ oxidizes on the surface of Ni through Ni peroxide formation (NiO_2_), recognized as O–O precursor. The results revealed the corrosion resistance and maximum selectivity of anode material for OER at high current density. The electrode's mechanical stability and corrosion resistance have been attributed to the absence of metal phase, high ohmic resistance, and chemical inertness of anode material. However, the high ohmic and voltage loss across the FTO–catalyst junction increases the charge transfer resistance and decreases the efficiency of hybrid material. Therefore, a high conducting substrate with chemical inertness toward Cl^−^ intermediate is maybe the selective option.

In another report, bilayer comprising Co‐doped, Mg‐intercalated MnO_2_ overlayer and Co(OH)_2_ underlayer‐modified FTO revealed high FE in simulated seawater.^[^ ^66^ ^]^ The experimental results indicated that the upper layer could efficiently shield the Cl^−^ diffusion and permit OER intermediate adsorption. At the same time, the Co(OH)_2_ underlayer acts as an adsorption site for water molecules. The characterization details demonstrated that the interlayer spacing between MnO_2_ layers provides diffusion pathways for water molecules to reach the underlayer active sites, where divalent Mg^2+^ ions repel Cl^−^ ions. DFT calculations revealed that Mg ions could shield the functional areas from Na ions due to their size and structure. Figure 10c shows the octahedral geometry of [Mg(H_2_O)_5_(OH)]^+^ with and without Cl interaction via H bonding. Na ions have a smaller size and have more affinity to Cl^−^ ions than Mg ions.

The most common parameters to evaluate the corrosion resistance of materials are corrosion potential and current. Corrosion potential is the thermodynamic value, while corrosion current is the kinetic parameter representing the corrosion rate. A catalyst with high corrosion resistance exhibits high corrosion potential and a small corrosion current. Ren and co‐workers probed the influence of B modification in cobalt‐iron layer double hydroxide (Co_2_Fe LDH).^[^ ^73^ ^]^ No noticeable structural collapse or corrosion pitting has been observed on catalyst surface after 28 days of immersion experiment, demonstrating that B modification substantially improves the structural resistance of the material. The controlled corrosion experiments showed that B modification reduces the corrosion current value from 2.4 to 1.38 μA cm^−2^, while the corrosion potential value increases from −0.321 to 0.388 V (vs. standard hydrogen electrode (SHE)). B modification increases the corrosion resistance and selectivity of anode material direly needed for practical applications.

Transition metal nitride (TMN) has high mechanical stability, optimized electronic structure, and high corrosion resistance considered a promising candidate for seawater splitting.^[^ ^74^, ^75^ ^]^ 3D core–shell TMN comprises Ni–FeN‐decorated Ni–MoN nanorod that has been reported for seawater electrolysis that shows high corrosion potential at high TOF in aggressive seawater electrolysis. The in‐depth investigations unveiled that the Ni–Fe N structure chemically oxidizes to Ni–Fe oxy/hydroxide with more exposed electrophilic Ni and Fe sites, increasing OER selectivity and pushing the Cl^−^ ions from the anode surface.^[^ ^76^ ^]^

Recent findings demonstrated that carbon‐based materials have high corrosion resistance compared with TM. Lee and co‐workers used a unique approach to increase the resistance of anode against Cl^−^ corrosion.^[^ ^77^ ^]^ Benefiting from the high electrical conductivity, gas diffusivity, and Cl^−^ corrosion resistance, the graphene oxide layer has been used as a protective layer on the catalyst's surface (Fe@Ni–Co@NF). In‐depth analysis revealed that graphene oxide coating was responsible for the gas diffusion and high Cl^−^ corrosion during seawater oxidation. The tinier interlayer spacing decreased the intercalation of Cl^−^ ions during OER in seawater electrolyte and increased the Cl^−^ corrosion resistance.

Esposito and coworkers investigated the role of silicon oxide overlayer on Pt electrode to block the Cl^−^ ions diffusion and adsorption on the electrode surface.^[^ ^78^ ^]^ The experimental results revealed that the ultrathin Si–O layer considerably enhances selective mass transport and suppresses Cl^−^ oxidation in simulated seawater. Mass transfer limiting current densities characterization showed that Cl^−^ permeability over Si–O layer is three orders of magnitude smaller than that of conventional membrane used in reverse osmosis process. The authors claimed that OER/Cl^−^ transport selectivity could be further increased by optimizing the structure and composition of the overlayer. The Cl^−^ repulsion can be further improved by integrating this overlayer with anionic fixed charges that electrostatically repel the Cl^−^ ions.

### 4.4 Amorphous Structure

As discussed in the introduction, insoluble precipitate formation and different microbes (bacteria) in actual seawater block the active sites and significantly suppress the OER kinetics. Research findings demonstrate that this problem would be fixed by designing the electrocatalyst with a large surface area and exposed reactive sites. Many studies are endorsing the efficiency improvement in seawater electrolysis while using amorphous electrode materials. Amorphous materials have a unique feature of structural flexibility which grants them to modulate the structure according to reaction conditions and offer means for both surface‐ and volume‐confined electrocatalysis. The other special features of amorphous materials which make them promising for natural seawater electrolysis are listed. 1) Amorphous materials have a high specific and electrochemically active surface area per geometric mass. 2) More incredible electrode–electrolyte interface due to super hydrophilicity as electrolyte diffuses into the inner part of the structure. 3) The self‐healing property of amorphous materials demonstrates high corrosion resistance and ensures the long‐term durability of active sites in aggressive seawater. 4) Ease in volume and surface reconstruction and possess high structural stability. 5) Amorphous materials could be easily transformed to partially crystalline material to expose the amorphous–crystalline interface to tune reaction intermediates’ adsorption energy. 6) The highly defective structure possesses more active sites with high intrinsic activity.

**Figure 11:**
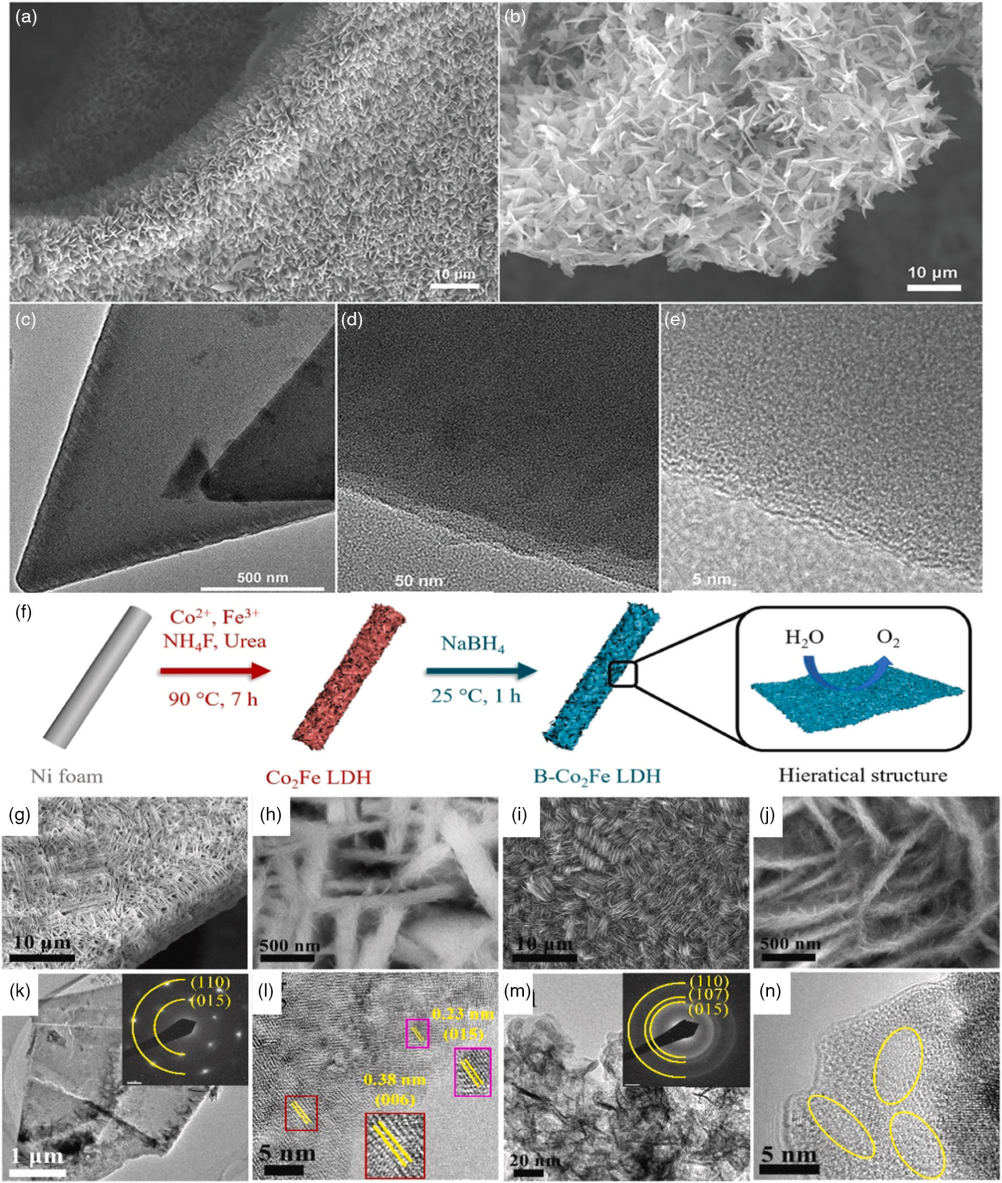
SEM images of a) Ni foam‐supported Co‐ZIF/L precursor and b) Ru‐incorporated amorphous Co oxide (Ru–Co–O_*x*_/NF). c–e) TEM images of Ru–Co–O_*x*_/NF. Reproduced with permission.^[^ ^80^ ^]^ Copyright 2021, Wiley‐VCH. f) Systematic Illustration of the formation of partially amorphous B–Co_2_Fe–LDH comprising two steps of water bath reaction followed by chemical reduction of the oxide with NaBH_4_. g,h) SEM images of Co_2_Fe–LDH, i,j) SEM images of B‐doped Co_2_Fe–LDH, k) TEM, l) HRTEM images of Co_2_Fe–LDH, and m) TEM and n) HRTEM images of B–Co_2_Fe–LDH. Reproduced with permission.^[^ ^73^ ^]^ Copyright 2021, Elsevier

It has been reported that surface and interconnected structures have a critical role in controlling the electrochemical response. The amorphous structure has abundant active sites and exposed facets and possesses more reactive centers.^[^ ^79^ ^]^ Wu et al. reported Ru‐modified amorphous cobalt oxide catalyst, which needed small overpotential to drive a high geometric activity.^[^ ^80^ ^]^ X‐ray diffraction (XRD) and high resolution transmission electron microscope (HRTEM) analyses validated the amorphous nature of the reported material (**Figure** **11**a–e). The catalyst sustains the high current density for more than 100 h revealing its long‐term durability. The high geometric activity and long‐term sustainable behavior were attributed to the amorphous structure that provides more active sites for seawater electrolysis. The inevitable precipitation suppresses the efficiency, but high electrochemical active surface area (ECSA) ensures the increased activity in seawater electrolysis.^[^ ^78^ ^]^

It is found that a broad interlayer spacing between LDH favors the diffusion of ions and increases the active sites’ infinity for OH^−^ ions. Tang and co‐workers revealed that the amorphous structure stabilizes the OER intermediate and provides reactive boundaries for electrochemical reactions.^[^ ^81^ ^]^ The amorphous structure inherently possesses more surface defects and roughness, uncoordinated sites, and disordered structure than its crystalline counterpart. Tu et al. investigated the structure effect for OH^−^ and Cl^−^ ions adsorption and hypothesized that OER catalytic efficiency in seawater depends on the exposed active sites preferentially adsorbing OER (OH^−^) intermediates.^[^ ^82^ ^]^ They synthesized two structures of Ni–Fe LDH crystalline structure with fewer boundaries, and the other was an amorphous phase. X‐ray analysis demonstrated that structure boundaries separated the crystalline and amorphous phases having more Ni^3+^ than other areas. Anion chromatographic analysis revealed that crystalline planes have a higher affinity for Cl^−^ ions while OH^−^ adsorb on amorphous and crystalline structures. It has been reported that amorphous facets are more active and show higher structural durability during OER in aggressive seawater. The authors used anion chromatography to probe the impact of the Cl^−^ adsorption on both the amorphous and crystalline structures. The results have shown that Cl^−^ adsorption decreases with increasing amorphous content due to its intrinsic nature as a soft basicity. The OH^−^ ions compete with Cl^−^ ions for reactive sites, abundantly present at interface structure (Ni^3+^). The OH^−^ is a harder base than Cl^−^, whereas Ni^3+^ is harder acid than Ni^2+^ and follows the hard soft acid base concept for adsorption. These results revealed the reason as to why OH^−^ selectively binds on Ni^3+^ sites compared with Cl^−^ ion. As the crystalline phases comprise Ni^2+^ predominantly they have more affinity for Cl^−^ adsorption. The results also show that OH^−^ adsorption and O–O coupling are limited at crystalline phases while amorphous abundant active sites trigger the OER kinetics. The surface modulation and in situ amorphous layer formation during oxidation activation is an effective route to increase the sustainability of electrodes. 3D heterolateral Ni_3_S_2_/Co_3_S_4_ (Ni–Co S) has been reported where in situ‐created amorphous oxyhydroxide layer increases the corrosion resistance, selectivity, and activity of OER.^[^ ^65^ ^]^ The in situ‐generated amorphous layer modulated the electronic and structural characteristics. It strengthened the collaboration of multiple functions, that is, the porous Ni–Co–S facilitates the formation of an amorphous layer and enlarges the ECSA. The self‐created amorphous layer has intense, intimate contact with underlying active sites and shows high mechanical and chemical stability. The amorphous structure has flexibility and promotes the formation of an active layer during catalysis. Ni–Fe borate amorphous layer on Ni–Fe LDH accelerated the O–O coupling, enabling the dynamic species generation. The active species has intimate contact with the amorphous layer and governs the chemical and mechanical stability.^[^ ^83^ ^]^

Recent findings show that surface texturing, defects construction, and metal doping increase the amorphic content and the ECSA, further accelerating the seawater electrolysis. Luo and co‐workers revealed that S incorporation in TMO increases the amorphous content, improving electrode materials’ selectivity, efficiency, and corrosion resistance.^[^ ^38^ ^]^ The catalyst sustains high current density (100 mA cm^−2^)for 1200 h in simulated seawater, and then performance gradually decreases to value with deep corrosion resistance and site blocking. The higher stability of electrode materials was assigned to an amorphous metal sulfide layer that prevents the active sites from pitting, erosion, and stress corrosion. At the same time, the enlarged surface area ensures high efficiency even after the physical adsorption of impurities. The post characterizations revealed that as the metal sulfide layer was partially destroyed, oxidative leaching of Cu–S occured in seawater. The insightful mechanism unveiled that chalcocite (Cu_2_S) was initially transformed to covellite (Cu–S) that was further oxidized and dissolves in aggressive seawater where Cl^−^ ions increase the dissolution rate and reduce the ASF. The following equations represent the dissolution mechanism of Cu_2_S (Equation ((24), (25), (26), (27), (28))).

(25) $${\text{Cu}}_{2}S+0.5O_{2}+2H^{+}\to ({\text{Cu}}_{2-x}S)\to \text{CuS}+{\text{Cu}}^{2+}+H_{2}O$$

(26) $$\text{CuS}+0.5O_{2}+2H^{+}\to {\text{Cu}}^{2+}+S^{0}+H_{2}O$$

(27) $$\text{CuS}+2O_{2}\to {\text{Cu}}^{2+}+{\text{SO}}_{4}^{2-}$$

(28) $$\text{CuS}\to e^{-}\to {\text{Cu}}^{+}+S^{0}$$

(29) $${\text{Cu}}^{+}+2{\text{Cl}}^{-}\to {\text{CuCl}}_{2}^{-}$$

These findings endorse the importance of the amorphous layer to prevent the pitting and erosion–corrosion and the metal dissolution to ensure long‐term durability in aggressive seawater.

The other chemical and structural advantages of amorphous catalysts include the fast rates for ion diffusion and abundant percolation pathways due to the loose packing of atoms and structural disorders. This disorder ensures fast electronic kinetics and high corrosion resistance by pushing the Cl^−^ ions and preventing its diffusion from electrolytes. Ren and co‐workers developed self‐supported amorphous B‐modified Co–Fe LDH that shows superb seawater oxidation activity and needs a small overpotential of 310 mV to drive a high current density of 100 mA cm^−2^.^[^ ^73^ ^]^ It has been noticed that NaBH_4_ treatment can lower the crystallinity of freestanding anode material. This partial amorphousness led to improved corrosion resistance and structural stability and was evidenced to work well in natural seawater (Figure 11f–n). Researchers demonstrated that amorphous content increases the hydrophilicity of active sites, which is generally helpful for electrolyte diffusion and gas desorption. The defective interfaces positioned in the amorphous–crystalline phase boundaries are highly reactive, and the amorphous structure increases the OH^−^ adsorption sites. The poststructural evaluation revealed some insoluble precipitates and pitting cracks on the catalyst's surface, but the overall design has been preserved, showing mechanical stability and corrosion resistance. This is inevitable to prevent the surface poisonousness of some portion of the catalyst due to the irreversible adsorption of insoluble residues. Therefore, a large surface area electrode with electrolyte diffusion capability is crucial to retain the catalyst contact with the electrolyte and continue the seawater electrolysis. We recently reported partially amorphous sulfur‐substituted copper oxide (S–Cu_2_O–CuO) nanoneedles as anode for natural seawater oxidation. The nanoneedles were directly grown on Cu foil in the mixture of KOH and NaBH_4_ solution.^[^ ^58^ ^]^ The mechanistic study revealed that NaBH_4_ releases H_2_ gas by decomposing into boric acid and reducing the metal. The reduction process has been controlled by increasing the solution's pH, increasing the activation barrier for H_2_ production from NaBH_4_, and inducing the amorphous structure formation. The amorphous nanoneedles offer space for surface and volume rearrangement, alter the chemical properties, enhance the ECSA and hydrophilicity which favor the seawater adsorption. The S substitution furtherer enhanced the amorphous phase, lost the atomic packing, and introduced the structural disorders that offer large ECSA and are critical for natural seawater electrolysis to continue the catalytic process in the presence of insoluble residues.

In another report, we have described the role of the amorphous Au–Gd–Co_2_B@TiO_2_ sheet for seawater electrolysis.^[^ ^41^ ^]^ We have observed that amorphous catalyst has higher specific activity, ECSA, FE, and TOF than crystalline material. Gd–Co_3_O_4_ has an amorphous phase synthesized by the electrodeposition approach, while NaBH_4_ treatment imparts further increment in the amorphous structure. BH_4_^−^ reacts with cobalt oxide releasing a massive quantity of H^−^ ions vigorously and decreasing the pH of the solution. As the surface of cobalt oxide reduces, the pH increases rapidly as now the primary reaction is the hydrolysis of NaBH_4_. Recent experimental findings demonstrated that B substitution in Co oxide structure has a linear relationship with sodium borohydride concentration. (Equation (30)).

(30) $$4{\text{BH}}_{4}^{-}+2{\text{Co}}^{2+}+9H_{2}O\to {\text{Co}}_{2}B+3B{\left(\text{OH}\right)}_{3}+12.5H_{2}$$

### 4.5 Porous Structure

In addition to the improvement in the electronic conductivity for rapid charge transfer, electronic modulation to selectively bind with OER intermediates, and enlargement of ECSA to expose maximum reaction sites to improve the geometric activity, the high mass diffusion rate within the material is imperative for preferable migration of OH^−^ ions and dynamic desorption of O_2_ to vacate the reaction site for incoming reaction substance. Porous materials have unique chemical and physical characteristics and provide more reactive sites and diffusion channels for mass transportation during electrolysis. Porous frameworks trigger the charge and mass transport and expose more active sites for intermediate adsorption. Porosity substantially improves the roughness factor defined as the ratio of ECSA to the electrode geometric area and bestows a large contact area to electrolyte and facilitates the reactant and product transportation. The other unique features of porous materials are as follows. 1) The pore skeleton offers pathways for electronic migration and decreases the charge transfer resistance. 2) The open cavities have sufficient contact with the electrolyte and mitigate the mass transport limitations.

**Figure 12:**
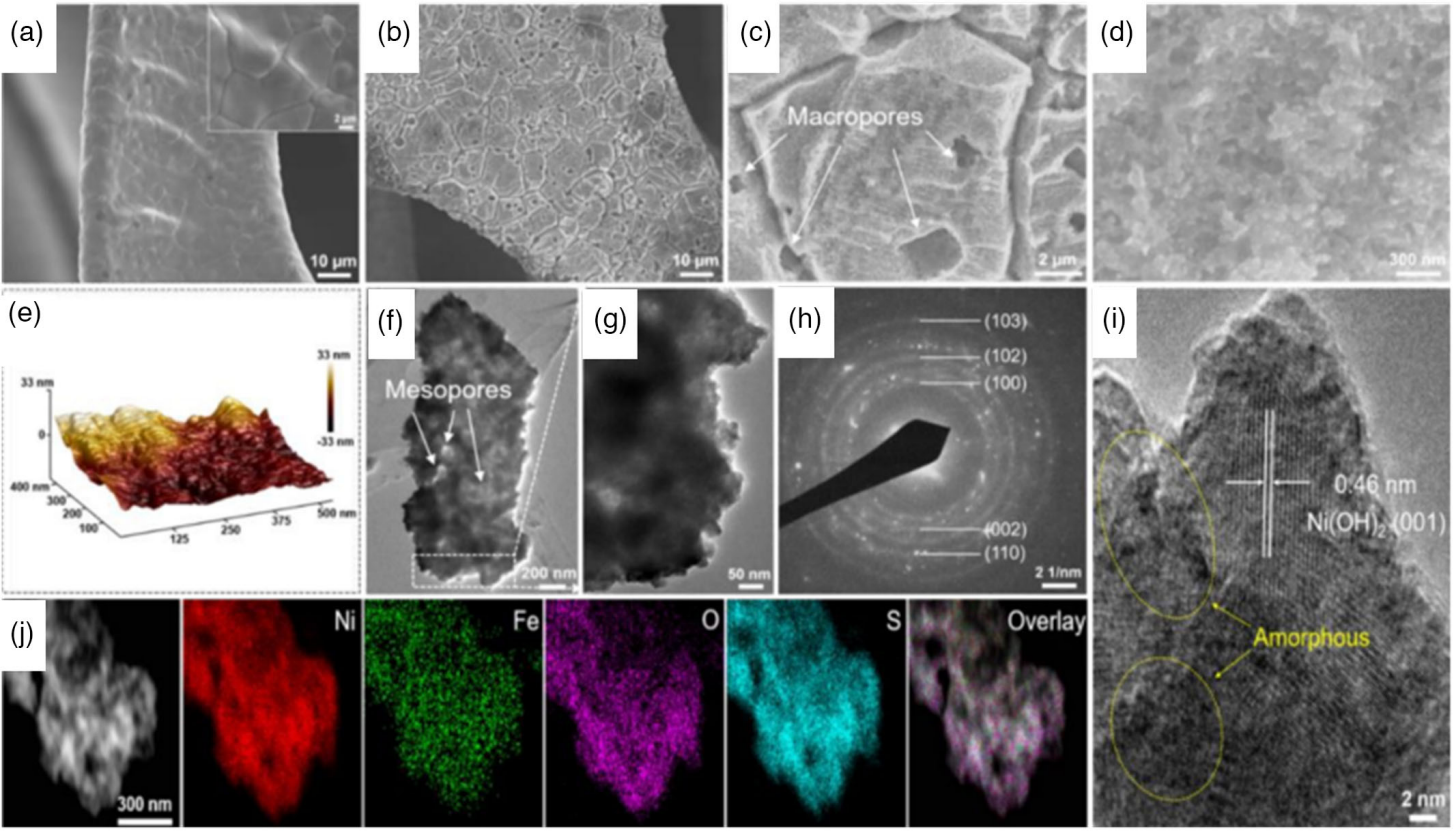
SEM images of a) bare Ni foam, b–d) S–(Ni, Fe)–OOH with different magnifications, e) surface topography, and f,g) TEM images, h) selected area electron diffraction pattern, i) HRTEM image of S–(Ni, Fe)–OOH, j) scanning transmission electron microscopy image, and elemental mapping. Reproduced with permission.^[^ ^84^ ^]^ Copyright 2020, Royal Society of Chemistry.

Ren and co‐workers synthesized porous S‐doped Ni–Fe–OOH directly grown on Ni foam at room temperature that needed a small cell voltage of 1.83 to drive a high current density (500 mA cm^−2^).^[^ ^84^ ^]^ The transmission electron microscopy (TEM) analysis revealed that oxyhydroxide particles are highly porous and comprise a mesoporous structure having pore size varying from 20 to 50 nm (**Figure** **12**). These tiny pores ensure the maximum exposure of reactive sites and accelerate the oxygen bubble detachment from the catalyst surface, highly valuable for seawater oxidation. The electrochemical response revealed that catalytic activity increases with time. This enhanced activity was attributed to the metal dissolution, which exposed more rough and porous active sites to OH^−^ adsorption. The postanalysis showed that mesopores provide an electrolyte transport channel and retain their surface texture after continuous operation in seawater electrolysis. The enhanced kinetics, structural stability, catalytic efficiency, and selectivity can be attributed to the following factors. 1)

The porous framework ensures the dense active sites and enormous ECSA for seawater oxidation. 2) The broad porosity distribution triggers the electrolyte diffusion and fast O_2_ detachments, both of which are needed for the high geometric activity. 3) Direct fabrication of Ni–Fe–OOH from Ni etching ensures the firm adhesion and intimate contact of active sites with current collector, reduces the charge transfer resistance, and supports chemical and mechanical stability at high current density.

Ren and co‐workers found that B modification through NaBH_4_ treatment created a porous structure and offered sufficient space for the diffusion and transportation of electrolyte and reaction intermediates.^[^ ^73^ ^]^ These porous structures favored gases detachment, mitigated the formation of a nonconducting layer on the catalyst surface, and increased the reaction durability. The same group synthesized the Ni–Fe LDH on Ni foam and demonstrated that the porous structure of the 3D Ni Foam network ensured the high loading of Ni–Fe LDH and exhibited the higher catalytic activity for seawater oxidation with negligible loss in efficiency after 96 h of continuous electrolysis at high current density.^[^ ^73^ ^]^ Similarly, Lv et al. synthesized porous feather‐like Ni–Co–P structures on Ni foam with visible holy structure promising efficient ionic transportation and desorption of gases molecules.^[^ ^85^ ^]^ The catalyst shows high activity and durability in natural seawater, attributed to the porous feather‐like structure. The insoluble residues have been observed on the surface of the electrode that can be removed by mild acid treatment to restore the catalytic efficiency, In addition, it was observed that open pores structures favor the removal of precipitates. Bimetallic phosphide Ni_2_P–Fe_2_P revealed high intrinsic activity and specific activity for alkaline seawater oxidation.^[^ ^70^ ^]^ The superior performance has been attributed to the ultrathin structure, optimized electronic configuration, and open porous structure that can offer adequate volume for mass transport and a fast rate of bubble detachment. Ren and co‐workers developed a 3D porous network composed of mesoporous Ni–Fe–N nanoparticles (NPs) interconnected and revealed a high diffusion rate TOF.^[^ ^76^ ^]^

**Figure 13:**
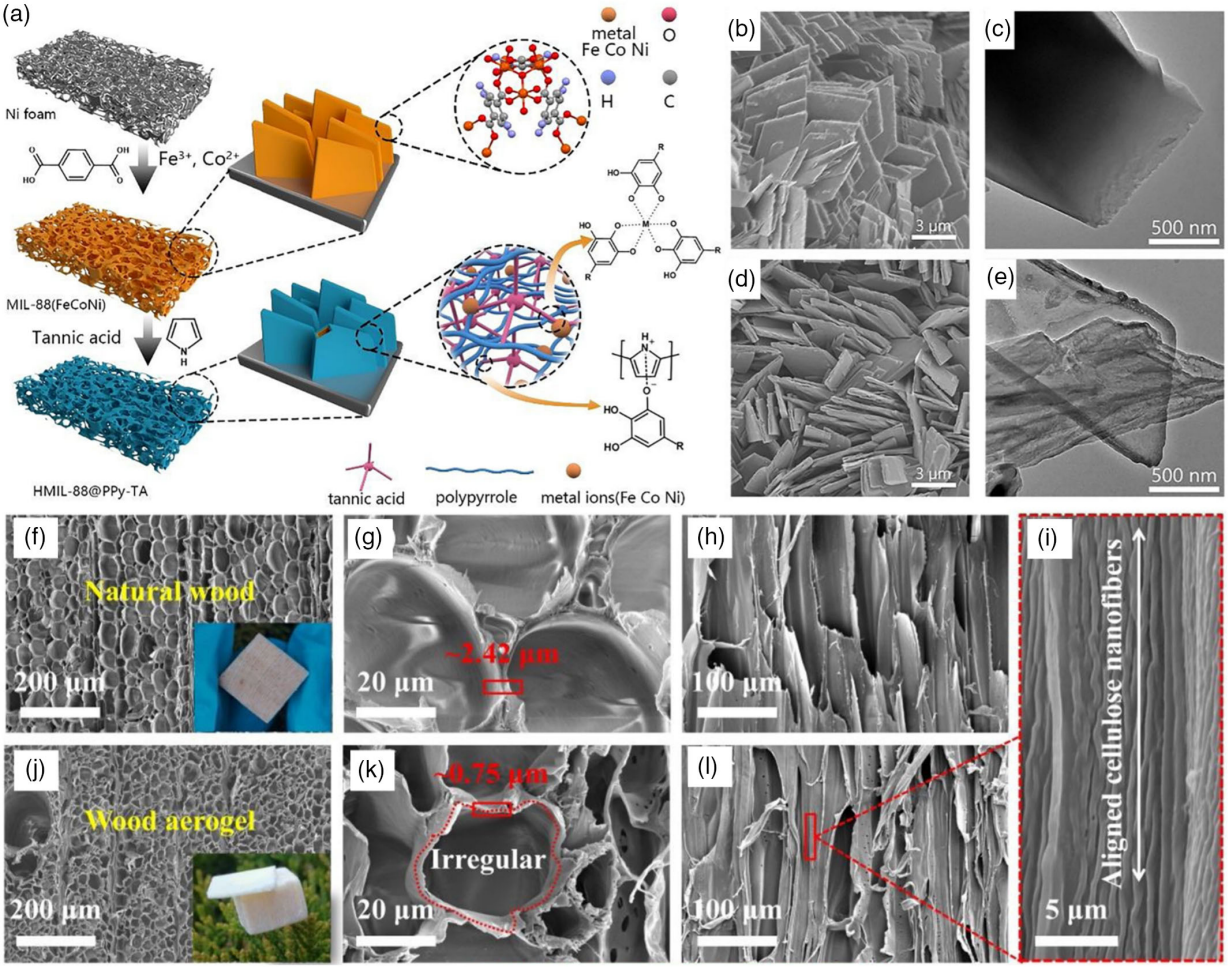
a) Systematic representation of the formation of the polypyrrole (PPy) and tannic acid (TA)‐modified hollow MIL‐88(Fe‐Co‐Ni) (HMIL‐88@PPY‐TA) on Ni foam, a). SEM images of b) MIL‐88(Fe‐Co‐Ni) c) and HMIL‐88@PPY‐TA d). TEM images of MIL‐88(Fe‐Co‐Ni) e) and HMIL88@PPY‐TA. Reproduced with permission.^[^ ^86^ ^]^ Copyright 2022, Elsevier. g,h) SEM images reveal the mesoporous structure of natural balsa wood with open and aligned microchannels, i–k) SEM images of wood aerogel with lamellar arch‐fashioned layer demonstrating the thinner thickness compared with natural wood. l) SEM images of well aligned cellulose nanofiber with rough and intricate structure. Reproduced with permission.^[^ ^88^ ^]^ Copyright 2021, Elsevier.

Metal–organic frameworks (MOFs) have been considered functional material to fabricate porous structures with enhanced ECSA and show outstanding ASF compared with conventional TMOs. However, in simple MOFs, water molecules could not diffuse properly due to special metal coordination and limited mass transport kinetics. The pyrolysis technique has been used to collapse the MOF coordination, resulting in exposure to more porous reactive sites to increase the ionic diffusion and gas desorption ability. However, pyrolysis is considered an energy‐consuming process and restricts the large‐scale application of MOFs. Chen et al. developed hollow trimetallic Fe–Co–Ni‐based MOF on Ni foam using tannic acid and poly‐pyrrole (**Figure** **13**a–e).^[^ ^86^ ^]^ The open structure allowed the catalyst reconstruction during electrolysis and showed remarkable corrosion resistance in seawater. Through surface reconstruction, the catalyst surface covered with active oxyhydroxide increases the activity and antitoxicity of active sites in Cl^−^‐containing electrolytes. Researchers unveiled that the porous structure defines the mass transport capacity and specific surface area of the electrocatalyst. 3D‐ordered porous structures with unique surface features maximize the exposed surface sites, permit the intermediate interaction with core active sites, endorse the electrolyte penetration to decrease the ion diffusion and electron transfer path, and pledge the fast mass transfer kinetics.^[^ ^87^ ^]^

The origin of overpotential is generally ascribed to the combined effect of ohmic voltage loss and mass transport limitations during the flow of charge. Therefore, nanoarchitectures, for example, low tortuosity, opened aligned channels, and highly porous structures, are desired to avoid the interfering ions and insoluble precipitates, facilitate gas desorption, and decrease the overpotential. Chen et al. developed sandwiches like S‐, P‐doped (Ni, Mo, Fe)–OOH/Ni–Mo–P/wood aerogel for seawater electrolysis.^[^ ^88^ ^]^ The catalyst has a high roughness factor, and hydrophilic features, allowing for induced porous structure and creating a firm interaction with guest materials (Figure 13f–l). The architecture comprises layer‐by‐layer assembling that stabilized the interfacial contact between heteroatom‐doped multimetal hydroxide and support and exhibited promising activity, selectivity, and durability in seawater electrolysis. The authors proposed the mechanism and attributed the activity enhancement to the induction effect of the template (wood aerogel). Figure 13 shows the sandwich‐like layered structure that offers a strong interfacial contact to Ni–Mo–P alloys. The high roughness surface with numerous hydroxyl groups ensured the high electron transfer rate at the interface. More significantly, the catalyst has direct and open microchannels, which are beneficial for electrolyte diffusion and permeation and increase gas desorption. It has been observed that initially when the catalyst triggered the seawater splitting, gases accumulated bubbles on the inner side of motivation. With continuous electrolysis, the bubbles size increases, and the whole bubble diameter approaches the channel size. The H_2_/O_2_ bubbles revealed the splitting phenomena due to the space limitation effect of channels, generating the bursting force, which pushes the insoluble residues away from the electrode surface and ensures the availability of OH^−^ intermediates’ activity sites.

### 4.6 Strong Catalyst–Support Interaction

The metal–support interactions define the steric environment, unsaturation, surface free energy, and chemical bonding of metal sites, thus leading to a different charge effect (charge transfer at interface and coordination of active metals with support), geometric effect (alteration in particles surface, geometry, and steric strain), and interfacial reactivity depending on the exposed interface.^[^ ^89^ ^]^ The metal support interface reshuffled the electronic density of both materials and changed the charge density of active sites. The energy band gap between the Fermi level of metal active sites and support determined the direction and magnitude of charge transfer. The support should have certain features, for example, defects, reducibility, exposed crystal planes, and conductivity, to reduce the charge transfer resistance.^[^ ^90^ ^]^ Markovic and coworkers suggested that the precise relation between activity and stability should be based on the ASF, defined as the ratio between the rate of O_2_ evolution and metal dissolution (Equation (31)).

(31) $$\text{ASF}=\frac{J-S}{S}$$

where *J* represents the O_2_ production in terms of current density and *S* shows the equivalent dissolution current density. The material with a higher ASF value at constant overpotential should be considered the best electrocatalyst for OER. They synthesized conductive nanoporous IrO_2_ shell on Ir core through fast dealloying of osmium from an Ir_25_Os_75_ alloy. The synthesized material reveals 8‐ and 30‐times improvement relative to dealloyed Ir_25_Os_75_ NPs and conventional iridium oxide, respectively. The improved stability was assigned to the dissolution of Ir, leaving only the most stable Ir‐oxide surface. The structural engineering of electrocatalyst through dealloying decreases the rate of dissolution and increases the ASF.^[^ ^91^ ^]^

The same group synthesized ultrathin oxide/hydroxide films on different TM substrates that show remarkable activity and stability for alkaline water splitting. In situ characterizations and DFT modeling were extensively used to investigate the stability of thin films. The underlying support radically stabilizes 2D nickel oxide/hydroxide, and the magnitude of stabilization depends on the adhesion energy and competition between intrinsic bulk stability. The same stability regime was observed for Mn and Co due to surface structure. The three‐phase boundary at the edge of Ni–OH in Pt‐supported Ni–OH film significantly reduces the activation barrier for OER through a bifunctional effect. The design principle for a highly stable catalyst was set by experimental and theoretical combo, which demonstrated that the careful selection of substrate, structural and compositional optimization of the host material, substrate and island size, and ad grain defects are the key controllers for the stability of electrocatalysts.^[^ ^92^ ^]^ The different parameters that affect the dissolution discrepancies are the catalyst loading, electrolyte flow rate, and presence of electrochemically dissolved metals species, binder content, and pH of the electrolyte.^[^ ^93^ ^]^ Chung et al. studied the activity–stability trends for OER on conductive M and Fe–M hydroxide/oxyhydroxide (M = Fe, Co, Ni) clusters. They revealed that Fe dissolution and redeposition rate at interface defines the stability of Fe active sites. The dynamic strength of active sites can be controlled by optimizing the Fe percentage in the electrolyte and its interaction with underlying support. It was concluded that chemisorption energy between Fe–M provides a reaction descriptor that affects the OER stability at the interface in an alkaline medium.^[^ ^94^ ^]^ The proper underlying support increases the reaction rate, and the strong metal‐support interaction increases the electrode material's chemical and mechanical stability. It has been explored that Au film as support plays a variety of roles in catalysis. In a recent report, Thomas Jaramillo and his coworker investigated that a higher amount of catalyst remained on Au substrate than other support.^[^ ^95^ ^]^ T. ul Haq et al. recently synthesized gold‐supported gadolinium‐doped cobalt boride amorphous sheets that show high activity and stability for OER. Being an excellent current collector, Au could consequently tune the inherent properties of the overall hybrid material. A minor positive shift was observed after 1000 cycles that mitigate the irreversible adsorption of impurities on the electrode surface. After long‐term electrolysis, the catalyst revealed a small charge transfer resistance to confirm the presence of a resistive‐free interface.^[^ ^79^ ^]^

In addition to noble metals, a transition metal substrate also serves as stable Skelton to decrease the surface energy of guest electrocatalyst, stabilize the active sites, and increase the mechanical resistance of electrocatalysts. Recently mesoporous Co foam‐supported Co_3_O_4_ nanowire was synthesized using one‐pot hydrothermal salinization of Co foam. The nanowires were chemically oxidized to Co–OOH, which shows superb durability for water splitting; no degradation was observed after 2000 h of continuous operation at high current density (100 mA cm^−2^). The outstanding stability was assigned to the following factors. 1) The electron density was transferred from high‐valance Co to Se center, and the strong hybridization between Se 4*p* and Co 3*d* electrons serves as a unique electronic coordination configuration for dynamic adsorption–desorption of intermediates. The electronic shift from the higher‐energy Co *d*‐orbital to lower‐energy Se *p*‐orbital and chemical transformation of Co to Co–OOH decreased the LUMO energy of Co–O intermediate and facilitated the nucleophilic attack of OH− ions for O─O bond formation. This orbital energy rearrangement ensures the sufficient contact of a reaction intermediate with conductive film and sustains the catalytic performance. 2)The deposition of Fe from the electrolyte due to dissolution of Co increased the ASF by increasing the O_2_ production. 3) The Co_3_Se_4_ nanowires were directly grown on Co foam due to dissolution–redeposition, which offers an intimate contact with the underlying support (current collector) and shows strong mechanical stability in harsh conditions. 4) The porous substrate framework triggers the electrolyte diffusion without bubble formation, provides an efficient conductive pathway to the electrolyte, and mitigates mass transport limitations. The formation of the oxyhydroxide layer ensures structural integrity.^[^ ^96^ ^]^

It has been investigated that the strong catalyst–support interaction stabilizes the OER intermediate on the active sites and reduces the activation barrier for H–O–H bond dissociation.^[^ ^97^ ^]^ Zhang and co‐workers investigated the impact of different conductive support on the OER activity of Ni–Fe LDH under identical conditions. The results revealed that rationally designed catalyst–support interactions dramatically impact the overall efficiency of the electrocatalyst. The Co‐supported Ni–Fe LDH has tenfold higher catalytic efficiency than that resulted from glass carbon, ITO, and FTO. The in situ findings revealed that highly charged Ni, Fe active sites with lower coordination, and shorter Ni, Fe─O bond lengths were only possible in Co‐supported catalysts.^[^ ^98^ ^]^

Ren and co‐workers reported the Ni–Co–N NP‐loaded nickel phosphide (Ni–P) microsheet and demonstrated that strong interaction between two phases ensured the efficient charge transfer at electrode and seawater electrolyte interface. The results revealed that Ni–P has high corrosion resistance and a robust skeleton to stabilize the host active sites. The same materials have been loaded on pristine Ni foam, which reveals poor corrosion resistance and inferior stability in seawater, endorsing that Ni–P support has much‐improved corrosion resistance.^[^ ^98^ ^]^ Enhancing charge transfer from conductive substrate to catalyst surfaces is an effective strategy to increase the reactivity of active sites for reaction and reduce the potential barrier at low overpotential.^[^ ^99^ ^]^

**Figure 14:**
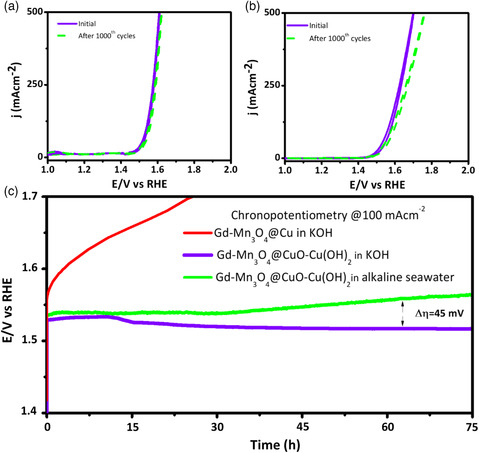
Gd–Mn_3_O_4_@CuO–Cu(OH)_2_ catalyst for seawater splitting. OER polarization curve recorded before and after 1000th cycles in a) 1 m KOH and b) 1 m KOH +Seawater. c) Durability test at constant current density of 100 mA cm^−2^ in alkaline seawater. Reproduced with permission.^[^ ^109^ ^]^ Copyright 2022, American Chemical Society.

The carbon support matrix has tunable surface chemistry and stabilizes the metal catalyst with high dispersion and corrosion resistance. Recently, researchers found that single‐walled carbon nanotubes (SWCNTs) induced the charge transfer from metal NPs to the surface and increased the electronic conduction.^[^ ^100^ ^]^ Substitutional doping regulates the intrinsic activity of the carbon matrix and decreases the charge transfer resistance between carbon and supported catalyst. N element is considered to be a potential dopant due to its pyridinic and pyrrolic chemistry, offering extra electrons to the carbon to retain the electronegativity of the matrix and induce a partial electrophilic center to accelerate the adsorption of OH^−^.^[^ ^101^, ^102^ ^]^ The N‐doped carbon anchored the catalyst, formed δ and π bonds with catalyst metal, and triggered the electron transfer rate between active sites and adsorbed intermediates, which is highly beneficial for the selectivity and efficiency of seawater electrolysis.^[^ ^103^ ^]^ It has been investigated that, in addition to N, electron donor S atom also tends to modify the π‐conjugated system of the carbon support. The S substitution enhanced the electronic density interaction between catalyst and support. It provided homogenously distributed anchoring sites to stabilize the small metal cluster, mitigating the aggregation and increased structural integrity.^[^ ^104^, ^105^ ^]^ The covalent bonding between S and C atoms created a large variety of metal species with ionic and metallic phases and increased the corrosion resistance of materials. Liu et al. investigated N/S‐codoped carbon nanosheets, which provide dispersed sites to stabilize the Ru NPs and increase the interfacial charge transfer. The doped carbon has a high surface area with porosity and homogenously stabilizes the small Ru NPs. The in‐depth investigation demonstrated that S incorporation into C‐based support promotes the electronic conduction between catalyst and support and increases the seawater dissociation kinetics.^[^ ^106^ ^]^ Zheng investigated that Ti‐supported bimetallic alloys have fast kinetics for seawater oxidation compared with unsupported alloys.^[^ ^107^ ^]^ The increment in catalytic activity was attributed to the new catalytic phase generation and the strong catalyst–support interaction. The results revealed that Ti support decreases the charge transfer resistance value, increases the electron lifetime, and enhances the M─O bond, promoting the OER intermediate adsorption. The strong metal interaction with underlying Ti support also increases the mechanical and chemical stability of active sites and governs a higher corrosion resistance during the electrolysis of aggressive seawater. Chi et al. developed a heterostructure of Fe–Ni(OH)_2_‐loaded Ni_3_S_2_ nanoarrays for seawater oxidation.^[^ ^108^ ^]^ The free‐standing anode needed small overpotential, charge transfer resistance, and Tafel slope value in seawater‐demonstrated favorable kinetics. This kinetic favorability was attributed to the lamellar edges of heterointerface and strong metal–support interaction between Ni_3_S_2_ and Fe–Ni (OH)_2_, accelerating the charge/mass transfer from the current collector to the catalyst surface. The controlled experiment has been conducted by loading the same amount of catalyst on bare Ni foam. The catalyst revealed comparable activity with noticeable degradation. These results endorsed the anticorrosive nature of Ni_3_S_2_ as a current collector. The Ni foam modification to Ni_3_S_2_ being a substrate reduces interfacial resistance and governs the structural and chemical stability of Fe active sites in seawater oxidation. In our recent report, we have modified the electronic and surface structure of Mn_3_O_4_ to increase the activity, selectivity, and corrosion resistance of active sites in unpurified seawater.^[^ ^109^ ^]^ The electronic structure was modulated by introducing the Gd impurity and creating the surface oxygen vacancies. The surface oxygen vacancies create the bandgap with unpaired *d*‐electrons, uplift the *d*‐state relative to Fermi level, and increase its interaction affinity with OER intermediate. The amorphous surface structure with microvoids ensures the proper contact between electrode‐electrolyte and accelerates the gases desorption as revealed from the high turnover frequency (150 s^−1^@1.55 V vs. reference hydrogen electrode [RHE]). The Cu substrate was modified to CuO–Cu(OH)_2_ nanostructure that provides high specific surface area and structural interaction for the growth of Gd–Mn_3_O_4_ nanosheets. The researchers observed that the catalyst directly deposited on Cu substrate loses its activity after a few cycles. However, after substrate modifications, the catalyst delivers a high geometric activity of 500 mA cm^−2^ at an input voltage of 1.63 V versus RHE and sustained this high current density for 75 h without noticeable degradation and hypochlorite formation (**Figure** **14**).

In addition to the substantial efforts in the rational design of anode, researchers have also focused on the engineering of electrolytes to increase the selectivity and corrosion resistance of anode in seawater electrolytes. Liu and coworkers used the common‐ion effect to suppress the solubility of NaCl into the electrolyzer. During this approach the concentration of NaOH was increased to 6 m which halved the NaCl saturation concentration, causing NaCl crystallization, mitigating the stress, and pitting corrosion caused by Cl^−^ anions.^[^ ^110^ ^]^ The researchers have used Ni–Co–Fe phosphide as both cathode and anode in the Ca/Mg‐free seawater electrolyte system and the catalyst sustains the high geometric activity of 500 mA cm^−2^ for 100 h. Similarly, the installation of Nafion membrane in electrolyzers increases the NaCl crystallization rate due to the ionic migration of Na ions from the anode toward the cathode.

## 5 Conclusions and Future Perspectives

Seawater electrolysis is a promising transformative technology for desalination, environmental remediation, and sustainable hydrogen production. The bottlenecks are: 1) the presence and interference of noninnocent impurities, especially Cl^−^ reactions, namely, hypochlorite formation and CER impeding the seawater splitting kinetics by competing with OER on the anode; 2) Cl^−^ ions’ high corrosivity that severely erodes the TM‐based catalysts, leading to poor catalyst durability and structural integrity; and 3) active sites that are blocked by dissolved ions, insoluble residues, bacteria, and other microorganisms, reducing the number of active sites and limiting mass transfer channels. In this review, from a perspective of practical applications, we recommend that it is essential to design the cost‐effective electrocatalyst by considering the high intrinsic activity, selectivity, and continuous operational capability at a high geometric activity. An ideal anode for seawater electrolysis should possess the following features simultaneously: 1) selective exposed active sites with high intrinsic activity; 2) high mechanical and structural stability and corrosion resistance; and 3) effective electron and mass transfer. Considering this, we have proposed rational engineering strategies and summarized the recent progress aiming for highly efficient and cost‐effective natural seawater electrolysis. The strategies are 1) electronic modulation of an electrocatalyst for optimizing active sites’ chemisorption energy to advance their selectivity for OER; 2) the creation of oxygen vacancies to modulate the surface structure, create new active phases, and increase the electron transfer; 3) a protective layer next to the OER active sites to prevent Cl^−^ from adsorption and increase the selectivity and durability of anode material; 4) amorphous structure for increasing the surface area, hydrophilicity, and self‐healing; 5) a porous structure to enhance the diffusion channels for mass transport and gas releasing to mitigate the catalyst degradation resulting from the blockage of active sites; and 6) optimized catalyst‐support interactions to lessen the leaching probability at a high current density where immense gas produced. These features should be incorporated into one electrocatalyst to ensure seawater electrolysis viable for massive H_2_ production.

Despite significant progress and recent research activities, the execution of direct seawater electrolysis is a long way ahead to achieve sustainable and affordable green hydrogen production. Some perspectives are mentioned from the aspects of advanced anode materials and electrocatalytic practices that can be implemented in future research.

Unfortunately, there are no established criteria for the catalyst evolution within the small seawater electrolysis research community. The catalytic performance of catalysts with the same structure and composition varies due to different working substrates, mass loading, pH of electrolyte, and preparation methods. Although Jaramillo and Horn's groups recommended some standard practices to evaluate freshwater's catalyst performance, seawater electrolysis needs more specifications.^[^ ^111^, ^112^ ^]^ We urged researchers to follow the following steps to evaluate the catalyst performance.

### 5.1 Activity

The catalytic activity in terms of high geometric activity is the primary figure of merit to screen the efficient catalyst. An efficient anode should deliver high current density at low overpotential. The catalysts should be evaluated and compared under industrially relevant conditions, that is, current density (>500 mA cm^−2^) at overpotential (<470 mV, concentrated alkaline seawater (6–10 m KOH) and at elevated temperature (60–80 °C).

### 5.2 Stability

The KOH addition increases the OH^−^ concentration that decomposes the bicarbonate ions in seawater to CO_3_^2−^ ions. These carbonate ions hydrolyze and can lead to precipitation of magnesium hydroxide (Mg(OH)_2_).^[^ ^112^, ^113^ ^]^The precipitates block the electrode surface and cause the mass and ion diffusion limitation. Usually, researchers filter these precipitates by centrifugation and report the catalyst stability in filtered seawater.^[^ ^48^ ^]^ Filtering out the residues is a convenient trick but not a standard protocol. The actual stability of the catalyst can be established when these sediments are considered. We encourage the researchers to report the durability of anode material in both filtered and natural seawater to validate catalysts’ antitoxicity and corrosion resistance. There is a probability that metal oxide and derivatives sometimes dissolve in the alkaline electrolytes at high anodic potential due to the high polar index value. For further justification, we recommended the ICP–OES analysis after continuous electrolysis.^[^ ^59^, ^114^ ^]^

### 5.3 Selectivity

FE calculation is crucial to determine the selectivity of anode material for OER at high current density. Although Cl^−^ oxidation is thermodynamically less favorable than OER and in basic solution, an anode can demonstrate a kinetic overpotential of 480 mV without any interfering chlorine chemistry. However, due to the four‐electron process, OER kinetics is much more sluggish than the two‐electron chemistry of the Cl^−^ oxidation. The FE at constant potential is different for different catalysts depending on the activity of the catalyst. Kweon et al.; determined the FE of carbon paper (CP), Pt/C, and Ru dispersed on MWCT (Ru@MWCT) in the range of 1.5–1.8 V.^[^ ^115^ ^]^ They experimentally observed that bare CP has a FE of only 11.4% at 1.8 V, and there was no FE below 1.7 V. The Pt/C electrode demonstrated Faradaic efficiencies of 46.99 and 85.97% at 1.5, and 1.8 V, respectively, while the Ru@MWCNT electrode revealed 85.88 and 92.28% at each corresponding voltage. The researchers are encouraged to report the FE in fresh and seawater at the same potential. In addition to the evolved‐gasses analysis, the possible presence of ClO^−^ products in the alkaline seawater electrolyte after the stability experiment should be analyzed using a colorimetric reagent. The color retention of electrolytes in the presence of the indicator and the absence of characteristic hypochlorite peak in UV/Vis spectrum mitigates the hypochlorite presence in the electrolyte.^[^ ^32^ ^]^

### 5.4 Corrosion Resistance

Researchers are encouraged to record the potentiodynamic polarization curves for their catalysts and determine the corrosion potential and corrosion current. Corrosion potential is the thermodynamic value, while corrosion current is the kinetic parameter representing the corrosion rate. A catalyst with high corrosion resistance exhibits high corrosion potential and a small corrosion current.

### 5.5 Postcatalyst Characterization

The most critical question is the configuration of the final state (i.e., postcatalyst). For example, some related references have demonstrated that TM may exhibit surface reconstruction to form the MOOH phase.^[^ ^116^, ^117^ ^]^ This is highly recommended to characterize the postcatalyst after seawater electrolysis. The structural characterization should be considered to demonstrate the structure collapse or corrosion pitting in aggressive seawater.

Although OER mechanism in a broad pH range has been reported, the precise and commonly accepted route for O–O coupling is still not apparent. In alkaline medium OER reaction on transition metal oxides and its derivatives (e.g., boride, nitride, sulfide, selenide) proceeds through multiple OH intermediates, that is, M–OH, M–O, MOOH, where M represents the active site. In literature, both single‐ and dual‐site mechanisms have been proposed, depending on the O─O bond‐forming step.^[^ ^27^ ^]^ The single‐site mechanism involves one active metal site where an OH attack on the MO intermediate forms MOOH intermediate while in dual‐site mechanism, two metal sites participate, and O─O bond formation is the combination of two M═O intermediates (lattice oxygen mechanism). Recent findings demonstrated the lower reaction energy barrier for the lattice oxygen route.^[^ ^118^ ^]^ Unfortunately, lattice oxygen participation in O–O coupling triggers the metal dissolution and decreases the ASF.^[^ ^119^ ^]^ Recent findings also demonstrate that if O─O bond is formed through a bifunctional mechanism that supports the direct formation of O_2_ through OH^−^ nucleophilic attack coupled with a concerted hydrogen transfer to a neighboring acceptor site, the overpotential might be further reduced (Equation (32)).^[^ ^120^ ^]^

(32) $$M=O+{\text{OH}}^{-}+A\to M+O_{2}+A-H+e^{-}$$

However, it is still ambiguous that which OER mechanism is most favorable, especially in seawater electrolysis. DFT calculations should be used to interrogate the optimum mechanism of seawater oxidation by accounting for the adsorption energy difference between reaction intermediates (OER and hypochlorite formation or CER) and active sites.

Though it is mainly believed that in situ‐formed oxide/hydroxides during OER are the active sites, other works revealed that catalyst offers an active phase. In the case of oxide derivatives, for example, boride, sulfide, nitride, etc., the question is whether the original compound offers an active center for intermediate adsorption or in situ‐formed oxide/hydroxide. The in‐depth in situ and operando characterization techniques should be used to probe the key intermediates, preferred reaction pathways, and actual active sites. Recently researchers have explored different operando techniques, including X‐ray absorption spectroscopy,^[^ ^121^ ^]^ XRD technique,^[^ ^122^ ^]^ Fourier‐transform infrared spectroscopy,^[^ ^123^ ^]^ and Raman spectroscopy;^[^ ^124^ ^]^ however, more investigation is needed.

Researchers have established diverse strategies to increase the number of active sites and intrinsic activity of each active site, such as doping, alloying, heterojunction, defects, and strain engineering. Despite the outstanding achievements, many Earth‐abundant TM electrocatalysts suffered from limited activity and selectivity in seawater electrolytes.^[^ ^125^ ^]^ Scalable and innovative strategies using advanced technologies such as machine learning and material genome to engineer the surface, electronic, and crystalline structure to increase the selective active sites for OER, enhance the ECSA, charge and mass diffusion, and gas detachment would be desirable to meet the needs for practical catalysts. We speculate the following anticipation and strategies. 1) The recent finding demonstrates that interface structure between two active sites with different chemisorption energy can be used as a channel for electron transportation through various intermediates.^[^ ^126^ ^]^ The physical and chemical properties of catalysts can be controlled by controlling the surface and the structure of different catalysts at interface boundaries.^[^ ^127^ ^]^ The size alteration of a component at the interface modulates electronic structure for optimizing proton and electron transfer and balancing the intermediate adsorption–desorption with optimum on the surface and interface of the catalyst.^[^ ^128^ ^]^ The size reduction at the interface provides a way to tune the chemisorption behaviors for affecting the reactant reactivity. The decrease in particle size at the interface creates a strong interfacial interaction and promotes the electronic conductivity, changes the BE for intermediate, and improves the catalytic activity, stability, and selectivity of electrocatalyst.^[^ ^129^ ^]^ 2) Transition metals are highly active and potential contenders for OER but nevertheless highly needed to improve their conductivity, activity, and electronic structure for enhanced catalytic potential. It has been extensively explored that some precious metals (Ru, Ir, Au) ameliorate the performance of transition metal‐based catalysts due to the electronic structure modification of active sites. However, these metals are precious and limited to large‐scale applications. In this regard, the ultralow content of precious metals is one of the promising approaches. The single‐atom catalyst loading is one of the potential strategies to minimize the usage of precious metals while loading many cost‐effective metals. Suppose this hybrid material possesses high TOF, FE, and durability in aggressive seawater. In that case, using a small area of such an efficient catalytic system is better than simple transition metal with a high geometric area and 3) compared with powder materials, free‐standing electrodes directly grown on the current collector are more promising for seawater electrolysis. The binder‐free catalyst has more accessible active sites and preferably adsorbs the OER intermediates. The direct growth creates strong catalyst support interactions, decreases the interfacial charge resistance, and increases active sites’ structural, chemical, and mechanical stability. In free‐standing electrode surface engineering is easier to increase the super hydrophilicity–aerophobicity to enhance the water contact, trigger the gas detachments, and promote the charge and mass transfer (**Table** **1**).

**Table 1:** Reported seawater OER electrocatalysts

Catalyst/supporta)	Synthesis method	Structure	Passive layer	OER potential [V]	*C* dl [mF cm^−2^]	Electrolyte	References
Ni–Fe LDH/NF	Microwave assisted	Crystalline	–	*η*_187_ = 0.37	–	1 m KOH + 0.5 m NaCl	[15]
Ni–Fe LDH/GC	Solvothermal	Crystalline	–	*η*_10_ = 0.359	–	1 m KOH + 0.5 m NaCl	[30]
Ni–Fe–B_*x*_/Ni–Fe	Solid state	Amorphous	Ni–Fe–B_*x*_	*η*_100_ = 0.37	0.371	Simulated seawater	[32]
Cobalt carbonate hydroxide	Hydrothermal	Crystalline	–	*η*_50_ = 0.4		0.1 m NaOH + 0.5 m NaCl	[33]
Pb_2_Ru_2_O_7_–*x*/GC	Wet chemical synthesis	Crystalline	–	*η*_10_ = 0.21	–	1 m KOH + 0.6 m NaCl	[34]
NiFe–NiS_*x*_–Ni/NF	Electrodeposition	Amorphous	S–O_*x*_^−^	*η*_400_ = 0.300	–	1 m KOH + 0.5 m NaCl	[37]
Au–Gd–Co_2_B/TiO_2_	Electrodeposition	Amorphous	B–O_*x*_	*η*_500_ = 0.33	28	Alkaline seawater	[41]
Fe_2_O_3_–NiO/NF	Heating treatment	Crystalline	–	*η*_1000_ = 0.252	14.49	1 m KOH + 0.5 m NaCl	[45]
Co–Fe_2_P/NF	Hydrothermal	Amorphous	*P*–O_*x*_	*η*_100_ = 0.266	50.4	Simulated seawater	[46]
Na_2_Co_1−*x*_Fe_*x*_P_2_O_7_/CC	Sol–gel	Crystalline	P2O7	*η*_10_ = 0.35	2.12	Alkaline seawater	[48]
Fe–P–NiSe2/CC	Electrodeposition	Crystalline	*P*–O	*η*_500_ = 0.32	1.29	Alkaline seawater	[49]
Co−Fe−O − B/GC	Hydrothermal	Amorphous layer	oxy‐boron species	*η*_1000_ = 0.434	20.6	1 m KOH + 0.5 m NaCl	[57]
S–Cu_2_O–CuO/Cu foil	Anodization	Amorphous	–	*η*_500_ = 0.42	30	Alkaline seawater	[58]
Ni–Fe–OH/Ni–Fe	Acid corrosion	Crystalline	–	*η*_100_ = 0.39	324	Alkaline seawater	[62]
Ni2P–Fe2P/NF	Etching growth	Crystalline	M–P	*η*_500_ = 0.44	12.3	Alkaline seawater	[70]
S, B‐(Co‐Fe Cr)OOH/GC	Solution combustion	Crystalline core@ Amorphous shell	S–O_*x*_^−^ and B–O_x_^−^	*η*_10_ = 0.198	42.3	1 m KOH + 0.6 m NaCl	[71]
S, B–(Co–Fe–V)‐OOH/GC	Solution combustion	=	S–O_*x*_^−^ and B–O_*x*_^−^	*η*_10_ = 0.218	35.9	1 m KOH + 0.6 m NaCl	[71]
NiO/FTO	Spray pyrolysis	Amorphous	FTO	*η*_100_ = 0.35	–	1 m KOH + 0.5 m NaCl	[72]
B–Co2Fe LDH/NF	Wet chemical reaction	Partially amorphous	B–O_*x*_	*η*_500_ = 0.376	8.1	Alkaline seawater	[73]
NiMoN@NiFeN/NF	Hydrothermal	Amorphous	Nitride	*η*_500_ = 0.347	238.7	Alkaline seawater	[76]
GO@Fe@Ni–Co/NF	Electrodeposition	Amorphous	GO	*η*_1000_ = 0.345	–	1 m KOH + 0.5 m NaCl	[77]
Ni–Fe LDH/NF	Hydrothermal	Partially amorphous		*η*_500_ = 0.257		1 m KOH + 0.5 m NaCl	[82]
S–Ni–Fe–OOH/NF	Solution phase method	Amorphous	S–O_*x*_^−^	*η*_500_ = 0.398	2.75	Alkaline seawater	[84]
Ni–CoP/NF	Hydrothermal	Crystalline	P–O	*η*_10_ = 0.28	19.7	Alkaline seawater	[85]
Co–Se/Co foam	Hydrothermal	Crystalline	–	*η*_100_ = 0.28	80.2	Alkaline seawater	[130]
Pt–Pd/Ti	Electrodeposition	Crystalline	–	*η*_10_ = 0.77	–	Seawater	[107]
Co–CoO–Rh/Cu foam	Wet chemical reaction	Crystalline	–	*η*_10_ = 0.26	5.13	1 m KOH + 0.5 m NaCl	[131]
NiO–Fe_3_O_4_@g–C_3_N_4_	Microwave Assisted	Amorphous		*η*_10_ = 190	36.5	Alkaline Seawater	[132]

a) NF = Nickel Foam, CC = Carbon cloth, GC = glassy carbon, GO = graphene oxide.

## Conflict of Interest

The authors declare no conflict of interest.
